# Innovative approaches for managing patients with chronic vestibular disorders: follow-up indicators and predictive markers for studying the vestibular error signal

**DOI:** 10.3389/fresc.2024.1414198

**Published:** 2024-08-16

**Authors:** Frédéric Xavier, Emmanuelle Chouin, Brahim Tighilet, Christian Chabbert, Stéphane Besnard

**Affiliations:** ^1^ Sensory and Cognitive Neuroscience Unit LNC UMR 7231 CNRS, Aix-Marseille University, Marseille, France; ^2^ Pathophysiology and Therapy of Vestibular Disorders Unit GDR 2074, Aix-Marseille University, Marseille, France; ^3^ UNICAEN, INSERM U1075, COMETE, Normandie Université, Caen, France

**Keywords:** integrative vestibular rehabilitation, visual fusion, vestibular error signal, sensori-perceptual-motor system, monitoring indicators, predictive markers

## Abstract

**Introduction:**

Despite significant advancements in understanding the biochemical, anatomical, and functional impacts of vestibular lesions, developing standardized and effective rehabilitation strategies for patients unresponsive to conventional therapies remains a challenge. Chronic vestibular disorders, characterized by permanent or recurrent imbalances and blurred vision or oscillopsia, present significant complexity in non-pharmacological management. The complex interaction between peripheral vestibular damage and its impact on the central nervous system (CNS) raises questions about neuroplasticity and vestibular compensation capacity. Although fundamental research has examined the consequences of lesions on the vestibular system, the effect of a chronic peripheral vestibular error signal (VES) on the CNS remains underexplored. The VES refers to the discrepancy between sensory expectations and perceptions of the vestibular system has been clarified through recent engineering studies. This deeper understanding of VES is crucial not only for vestibular physiology and pathology but also for designing effective measures and methods of vestibular rehabilitation, shedding light on the importance of compensation mechanisms and sensory integration.

**Methods:**

This retrospective study, targeting patients with chronic unilateral peripheral vestibulopathy unresponsive to standard treatments, sought to exclude any interference from pre-existing conditions. Participants were evaluated before and after a integrative vestibular exploratory and rehabilitation program through questionnaires, posturographic tests, and videonystagmography.

**Results:**

The results indicate significant improvements in postural stability and quality of life, demonstrating positive modulation of the CNS and an improvement of vestibular compensation.

**Discussion:**

Successful vestibular rehabilitation likely requires a multifaceted approach that incorporates the latest insights into neuroplasticity and sensory integration, tailored to the specific needs and clinical progression of each patient. Focusing on compensating for the VES and enhancing sensory-perceptual-motor integration, this approach aims not just to tailor interventions but also to reinforce coherence among the vestibular, visual, and neurological systems, thereby improving the quality of life for individuals with chronic vestibular disorders.

## 1 Introduction

### 1.1 Background and justification for the study

Chronic vestibular disorders (CVS) manifest through nonspecific symptoms such as imbalances, blurred visions perceived during self or environmental movements, and disturbances in perception or even spatial memory. They pose a significant clinical challenge affecting a broad segment of the population (1, 2). While peripheral causes of these disorders are often identified initially, the impact of these peripheral impairments on the central nervous system (CNS), especially on regions associated with the vestibular system mainly multisensory related functions of the temporoparietal cortex, remains under-explored in clinical practice. This complex interaction highlights key questions about neuroplasticity of vestibular, visual and somesthetic integration, and the brain's adaptive strategies to sensory disturbances and vestibular rehabilitation techniques.

Nevertheless, the interactions and recurring complaints of visual disturbances noted in CPV are also questioned in the literature. Roberts et al. (3) highlighted a significant change in the primary visual cortex V1 in patients suffering from chronic vestibular neuritis during congruent visuo-vestibular stimulations. This discovery suggests that adaptive mechanisms associated with the primary visual cortex play a crucial role in central compensation and, by extension, in clinical outcomes in these patients. This observation is reinforced by Beh (4–7), who emphasizes the pivotal role of vestibular information in cognitive processes, particularly visuo-spatial abilities, and how vestibular disorders can lead to visuo-spatial deficits through lesions of cortical and subcortical components of the vestibular system. Finally, Cousins et al. (8) remind us that visual dependence are among the most important predictive symptoms of chronicity.

Xavier (9, 10) proposes considering the disruption of the integration of the peripheral vestibular error signal (VES), especially at a subliminal threshold level, which could influence, on one hand, short and medium-term visuo-oculomotor adaptations, and on the other hand, neuronal plasticity and the establishment of optimum compensation processes following a VES experienced by the CNS in the long term. At this stage, it's important to understand that visual fusion is a complex process that allows the human brain to combine images from both eyes into a single coherent three-dimensional image. This phenomenon, crucial for spatial perception, relies on adherence to two fundamental concepts: the horopter and Panum's area. However, this visual synergy can be compromised under pathological conditions, especially in the context of vestibular asthenopia (11). The horopter is a geometrical construct that defines the region of space where images projected onto the retinas of both eyes overlap exactly, ensuring normal retinal correspondence and optimal binocular vision for fusion and stereoscopic vision. Any deviation from this alignment leads to a discrepancy from the horopter, resulting in a perception of an image without relief, blurred, or in extreme cases, double. Panum's area, also known as the “fusion zone,” is the area around the horopter where binocular fusion is still possible despite slight discrepancies between the retinal images (12). This area plays an essential role in three-dimensional perception, as it allows for some tolerance to variations in the position of the observed object. We have demonstrated that a vestibular error signal (VES) can result in a subtle adaptation of oculomotor behavior involving an anomaly in retinal correspondence. This manifests as symptoms such as visual fatigue, blurred vision, and in extreme cases, intermittent diplopia, particularly when the fixation object moves or when the individual is subjected to complex body movements. This condition is referred to as vestibular asthenopia.

It is within this research context around CPV that we conducted a retrospective study at a physiotherapy center in partnership with the Caen Hospital Center.

### 1.2 Vestibular error signal and research hypothesis

The “vestibular error signal” (VES) refers to a discrepancy between expected sensory information and that perceived by the vestibular system, which plays a crucial role in maintaining balance and spatial perception. This gap can result from damage or dysfunctions at the level of peripheral or central components of the vestibular system. This concept, already present in the literature of the 1980s (13) has been enriched by numerous works done both in engineering and in the human model. Mathematical models of signal integration have allowed us to better understand the notion of error in measurement systems due to noise (unwanted signals), leading to differences and therefore errors between the output quantity and the input quantity to measure, especially in dynamic measurement situations where the mean squared errors take into account both dynamic and static errors (14).

#### 1.2.1 Multisensory interaction and central nervous system adaptation

These numerous observations in both fundamental and clinical research indicate that the vestibular system tends to interact with visual and somatosensory events. For exemple, Angelaki & Cullen (15) emphasized how vestibular signals contribute to an astonishing range of brain functions, from spatial perception to motor coordination. Chang et al. (16) examined how the integration of auditory and vestibular signals requires their simultaneous perception despite their asynchronous arrival at the central nervous system, proposing a mechanism to explain symptoms in patients with imbalance. Ferré et al. (17) demonstrated how vestibular stimulation differently modulates two sub-modalities of the somatosensory system, increasing touch sensitivity while reducing sensitivity to nociceptive inputs. The same authors in 2015 (18) showed how vestibular stimulation interacts with visual and somatosensory events in a detection task, highlighting the vestibular role in regulating somatosensory gain.

#### 1.2.2 Visuo-Vestibular integration and motor responses

Shayman et al. (19) explored the hypothesis that vestibular deficits could disrupt visuo-vestibular temporal integration, determining relationships between vestibular perception threshold and the temporal binding window in participants with normal and hypo-functioning vestibular function. In this context, the hypothesis of the VES playing a crucial role in maintaining certain subtle symptoms appears relevant. We know that the central nervous system processes discrepancies between expected movements and actual sensations. For instance, when a person moves or turns their head, the vestibular system anticipates changes in sensory perception based on the planned movements (15). If the actual sensory signals differ from these expectations, an error signal is generated. This error signal is then used to adjust motor responses and enhance the accuracy of future movements, as well as to update sensory perception and spatial representation (20, 21). But when facing a chronic peripheral VES, our hypothesis is that the mechanisms of sensory integrations and error signal processing are significantly altered. Alberts et al. (22) offer insights into how peripheral VES influences the noise levels of otolith and somatosensory signals depending on body tilt, leading to dynamic shifts in sensory input weights with tilt angle. This highlights a shift in sensory reliance, where otolith organs are more influential around upright positions, and somatosensory inputs become more critical at larger body tilts. Forbes et al. (23) further explored how peripheral VES affects motor responses, showing that it modifies the magnitude of muscle responses to align with the vestibular error and balance direction. This flexibility in motor command adjustments in response to vestibular disturbances points to the system's adaptability. Rideaux et al. (24) delve into the impact of peripheral VES on sensory integration, demonstrating how it leads to sensory reweighting and influences the activity balance between congruent and opposite neurons.

#### 1.2.3 Clinical implications and rehabilitation protocol

This affects the decision-making process on whether to combine or separate multisensory signals, underlining the brain's capacity to adapt to vestibular errors for precise motion estimation. This suggests that chronic peripheral VES not only disrupts sensory integration and motor response adaptation but also impacts the ability to manage visual-vestibular mismatches, potentially leading to headaches and dizziness. Thus, following a chronic VES, the necessary adaptations for navigating and effectively interacting with our environment would be poorly adjusted, and predictions and responses based on complex and often conflicting sensory information flows would be inadequate. This mismatch can lead to a variety of persistent symptoms in CVS, including, but not limited to, dizziness, instabilities, spatial disorientations, and difficulties in executing precise and coordinated movements. The impact of these alterations on patients' daily lives can be substantial, affecting not only their ability to perform ordinary tasks but also their psychological well-being. To address these observations, we undertook the creation of a vestibular rehabilitation program based on an integrative approach involving the clinical and instrumental identification of the type of VES (irritative or deficient) and the search for tracking markers dedicated to the type of VES. iVRT addresses vestibular disorders by considering the individual as a whole, including interventions on motor, oculomotor, cognitive, and emotional systems. In addition to vestibular exercises, the treatment incorporates the assessment and rehabilitation of the cervical spine to improve sensorimotor coordination, maxillofacial approaches to reduce muscle tension and enhance proprioception, and the learning of strategies to improve dynamic balance performance and stability. Neurovisual performance, which links vision and balance, is also a focal point, with specific rehabilitative sequences if anomalies are detected. Moreover, the approach addresses psychic and emotional aspects, recognizing the impact of cognition and emotional state on physical balance and utilizing psycho-behavioral assessment and management techniques (Table 1).

**Table 1:** Rehabilitation sequence.

Sequences	Title	Descriptions
1	Positional maneuvers	Traditionally indicated for resolving benign paroxysmal vertiginous events of peripheral positional origin. Also proposed in other settings for perceptual-sensory reweighting, notably in habituation.
2	Neurosensory reweighting	Tools and techniques aimed at creating a perceptual-sensory reweighting following a supraliminal vestibular stimulus that is incoherent.
3	Neurosensory facilitation	Tools and techniques designed to optimize the signal/noise filter by attenuating a vestibular error signal or through attentional tasks.
4	Sensory conflict induction	Tools and techniques aimed at increasing perceptual noise by artificially creating incoherence among sensory inputs.
5	Sensory integration optimization	Tools and techniques aimed at achieving a coherent response based on visual and motor context.
6	Perceptual-somatomotor and perceptual-visuo-oculomotor reweighting	In the presence of a vestibular error signal: tools and techniques aimed at optimizing perceptual-motor sensory integration through motor and/or sensory inputs to inhibit the integration of the vestibular error signal.
7	Gait and balancing performance	Tools and techniques aimed at physical conditioning and error experimentation.
8	Cognitive reweighting	Tools and techniques aimed at enhancing or optimizing cognitive-emotional and psycho-behavioral processes.

iVRT is structured around four main pillars: comprehensive evaluation of the patient's abilities and dysfunctions, personalized treatment, regular monitoring to adjust the treatment, and finally, the definition of termination criteria based on indicators of success or failure. The treatment sequences detailed in Table 1 are determined following the initial assessment.

**Figure 1:**
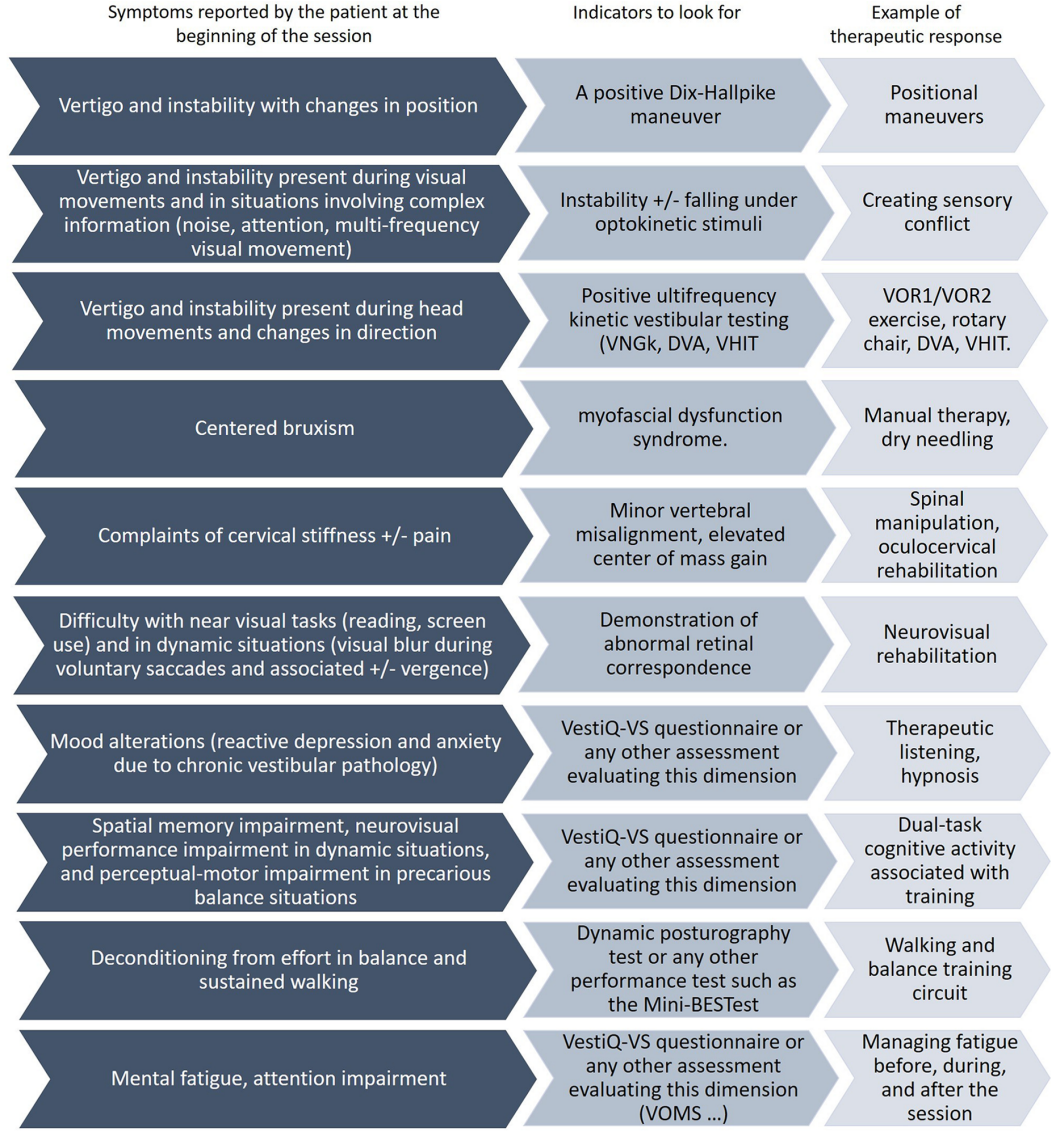
Decisional tree (Two parts). This figure illustrates the various symptoms reported by chronic vestibular patients. A comprehensive initial assessment is conducted at the beginning of treatment, and the main areas of focus are determined based on the most debilitating symptoms for the patient. At the start of each week, a screening of complaints (symptoms) is conducted. For each complaint, an evaluation is performed, and treatment is adjusted based on the results. VNGk: kinetic videonystagmography, DVA: dynamic visual acuity, VHIT: video head impulse test, VOMS: Vestibular Oculomotor Motor Screening.

“Each treatment session is customized according to two guiding principles: addressing the patient's specific complaints and being guided by specific instrumental indicators present in the literature for which we have constructed a decision tree Figure 1, (9, 10)."This strategy adheres to the diagnostic treatment model, ensuring a targeted and responsive approach to patient care. During this care, we searched for indicators related to statistically significant changes (monitoring indicators). Finally, we evaluated retrospectively whether there are predictive markers of postural instability and predictive markers of the variation in the accuracy and precision of the subjective visual vertical (25).

## 2 Materials and methods

### 2.1 Research objectives and study design

This retrospective study aims to identify monitoring and predictive markers in patients suffering from chronic unilateral peripheral vestibulopathy unresponsive to conventional therapies for more than a year. Conducted from November 2021 to March 2022, our research focuses on key indicators derived from questionnaires and instrumental evaluations to deepen the understanding of chronic vestibular pathology. The protocol was approved by the ethics committee of the Caen University Hospital, accreditation number 2,796, and was carried out in accordance with confidentiality and consent standards.

### 2.2 Materials and methods

#### 2.2.1 Participants

The study included patients with chronic unilateral peripheral vestibulopathy lasting one year or longer who had not responded to rehabilitative treatment. Rehabilitation follow-ups for these patients were conducted in a physiotherapy clinic specializing in vestibular rehabilitation located *in vitro*lles (13,127, France). To ensure the reliability and precision of the collected data, inclusion criteria were meticulously defined, relying on comprehensive clinical and instrumental evaluations.

Exclusion criteria were carefully chosen to eliminate any variables that could bias the study's results. These criteria included:

• Binocular or stereoscopic vision disorders: including neutralization, amblyopia (poor vision in an eye not corrected in childhood), anisometropia (difference in refractive power between the two eyes), and all types of strabismus, including microstrabismus, where the visual axes' misalignment is minimal but can affect depth perception.
• Psychiatric disorders diagnosed before the onset of vestibular issues to eliminate potential interferences from pre-existing psychiatric conditions that could influence vestibular symptoms or their management.
• Vascular, degenerative, and inflammatory neurological conditions affecting central functions diagnosed before rehabilitative care.
• Neurological conditions likely to impact the central nervous system and, consequently, confound the evaluation of peripheral vestibulopathy were excluded to purify the research sample from external influences that could alter the accuracy of the results analysis.

#### 2.2.2 Experimental procedures

Participants were evaluated before and after treatment using questionnaires (Supplementary Tables S1–S5) created from vestibular patient literature to assess i/handicap and quality of life: The Dizziness Handicap Inventory [DHI (26),], the Short Form (27) Health Survey [SF36 (28, 29);], ii/personality traits with the Big Five Inventory [BFI (30);]. Also included in our questionnaire battery were the 31-item Positive and Negative Emotionality Test [EPN31 (31);] and the Vestibular Health Questionnaire we developed (VestiQ-VS; Xavier et al. 2023 in submission). Additionally, a series of instrumental examinations included: i/a sensory organization test from posturography, developed by Synapsys, including a specific analysis called sensory organization assessment (Supplementary Table S6), ii/videonystagmography (VNG thermal and kinetic) developed by Synapsys, and the study of subjective visual vertical (SVV) iii/ the following optometry tests: for the evaluation of near and far visual acuity the use of Monoyer and Parinaud scales; for the evaluation of convergence and divergence capacity the prism bar (PB); for the evaluation of fusion the Mawas Board; for the evaluation of accommodation capacities [or near point of accommodation (NPA)] and convergence [or near point of convergence (NPC)] the use of the accommodation bar; for the evaluation of distant stereoscopic vision the Thomas Stereoscopic Vision Test (TVST); and for assessing a patient's degree of binocular vision and binocular single vision the Worth four light test; all these evaluations allowing an approach that encompasses somato-perceptual-visuo-oculomotor and somato-perceptual-motor aspects under the regulation of vestibular control (32–36).

#### 2.2.3 Indicators under study

A detailed analysis of the following indicators was performed:

##### 2.2.3.1 Synapsys posturography analysis

Sensory organization test (SOT) has an instrumental standard developed at the Cognitive Neuroscience Laboratory of St Charles Campus, Aix Marseille University. It is established from the Stability Limits and SOT conditions [Supplementary Tables S6 and S7 (27)].

The total energy calculation, assessing postural stability, is based on recording the trajectory of the center of pressure (CoP), representing the body's center of gravity movement on the support surface (37). The CoP speed is calculated in two directions (antero-posterior and lateral), yielding two data sets. The variance of these speeds is then calculated for each direction, and the total energy is obtained by combining these variances. A high total energy value indicates less postural stability, while a low value suggests better stability.

$$\varepsilon \mathrm{total}\;(mm^{2}.\;s)=\mathrm{Var}(\mathrm{APd})+\mathrm{Var}(\mathrm{MLd})$$

Where:

Var(APd) represents the variance of the CoP speed in the antero-posterior direction (APd),

Var(MLd) represents the variance of the CoP speed in the lateral direction (MLd).

##### 2.2.3.2 Kinetic videonystagmography (VNG) indicators

The model used includes a videonystagmography system and an electronic rotational chair (type Met4). We utilized the indicators obtained during the Met4 kinetic test in burst (sinusoidal test at 0.25 Hz) by studying the visuo-vestibulo-ocular reflex (test with the patient's eyes open without fixation; VVOR), the vestibulo-ocular reflex (test with the patient's eyes closed; VOR), the double-task vestibulo-ocular reflex [test with the eyes closed combined with a mental arithmetic task (random addition and subtraction including numbers between 1 and 100); VOR2], the ocular fixation index (test with visual fixation; OFI), and the cervico-ocular reflex [test with head stabilization (only the torso performs the sinusoidal movement); COR]. The standards are presented in the Supplementary Material, Table S8. The VNG Synapsys standards are norms developed by the manufacturer and are documented in the non-indexed internal technical documentation (38).

##### 2.2.3.3 Bithermal videonystagmography (VNGt) indicators

The indicators recorded during the bithermal test were the absolute nystagmic preponderance, the reflectivity on the side opposite to the lesion, and the ipsilateral deficit to the lesion. Norms are available in the Supplementary Table S8 (38).

##### 2.2.3.4 Composite indicator “state of compensation” (SoC)

We developed an indicator for this study to classify vestibular profiles via bithermal videonystagmography (VNGt), including: i/ Non-Inhibited Profile (N) with contralateral reflectivity ≥15°/s and ipsilateral vestibular deficit ≤ 30%, indicating preserved contralateral reactivity despite a minor deficit, thus without modulation of the subcortical arc; ii/ Partial Contralateral Inhibition Profile (P) when contralateral reflectivity is in the range [2°/s; 15°/s] with an ipsilateral deficit in the range [30%; 70%], showing partial compensation; iii/ Total Contralateral Inhibition Profile (T) defined by a reflectivity ≤2°/s and an ipsilateral deficit ≥70%, reflecting an almost total inhibition of contralateral peripheral input associated with maximum subcortical compensation; iv/Inhibition without Deficit Profile (I) with reflectivity ≤15°/s and an ipsilateral deficit ≤30%, indicating a reduction in contralateral reactivity despite a minor deficit. N indicates the absence of compensation modulation in the presence of a subliminal deficient-type VES, *P* indicates moderate compensation responding to a deficient VES, T indicates strong compensation responding to a deficient VES. I indicates modulation of reflectivity in the presence of a deficient VES maintained at a subliminal level. Reflectivity in vestibulometry refers to the reflex response generated by the vestibular system during bithermal caloric stimulation. Reactivity refers to the vestibular system's ability to respond to stimulation and modulate the signals sent to the brain. In the context of vestibulometry, reactivity is often assessed in terms of vestibular compensation following a loss or deficit.

##### 2.2.3.5 Composite indicator for the study of hyperactive signal (Hs)

**Figure 2:**
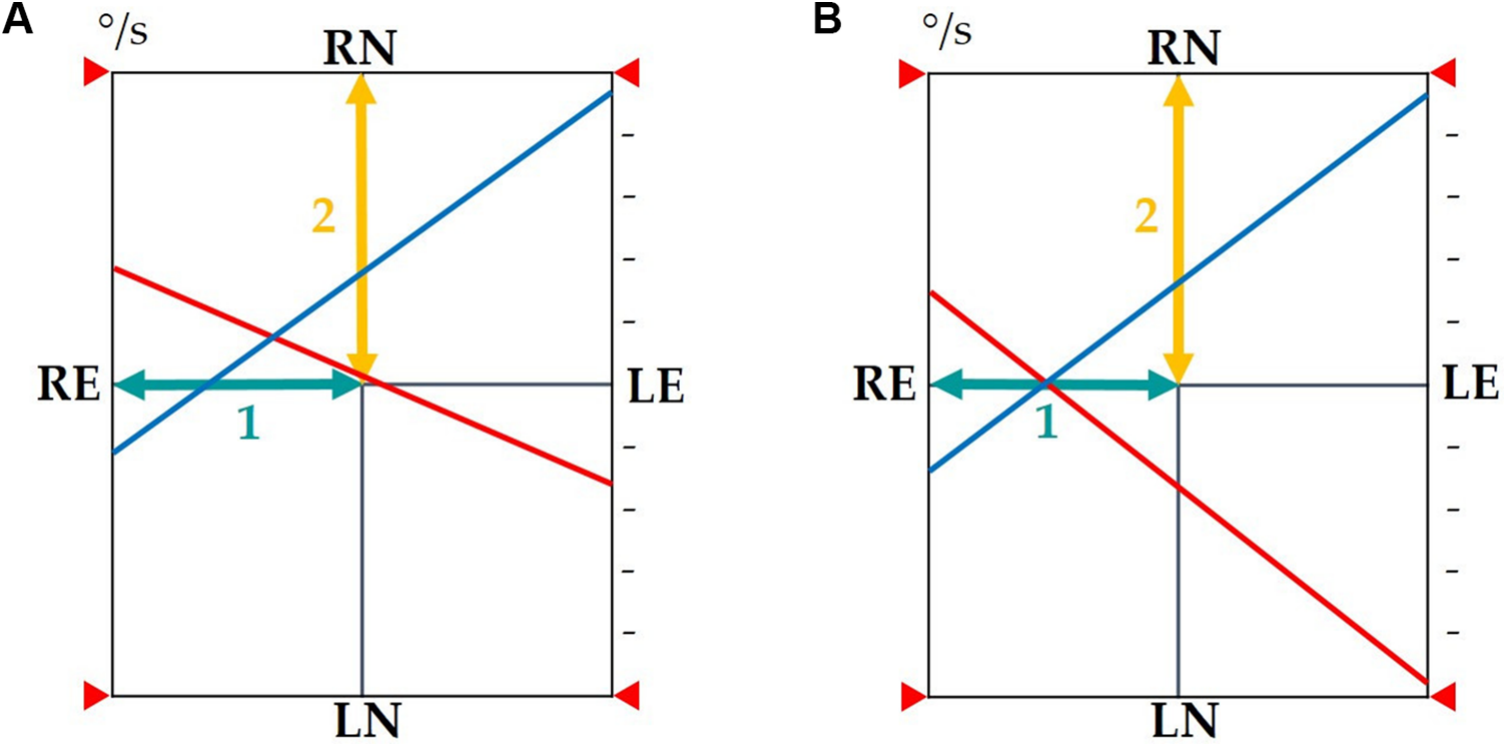
**(A)** In the presence of an uncompensated VES resulting from a subcortical compensation defect: The observed phenomenon will cause a shift in the intersection of the reflectivity lines along baseline 1 towards the pathological side and a shift in the intersection along baseline 2 upwards, which may indicate either an incomplete state of compensation of the vestibular nuclei during warm stimulation on the healthy side or a defect in reflectivity during cold stimulation on the pathological side. A revealed nystagmus beating towards the pathological side will be present (shift towards the upper left quadrant of the intersection point of reflectivity lines). **(B)** In the presence of a compensated deficient VES: The observed phenomenon will cause a shift in the intersection of the reflectivity lines towards the pathological side along baseline 1 without a parallel shift along baseline 2. The intersection of the reflectivity lines remains on the horizontal axis. A revealed nystagmus beating towards the healthy side will be present. VES, vestibular error signal; baseline 1, axis of directional preponderance; baseline 2, axis of reflectivities; red reflectivity line, results of warm stimulations of the right and left ears; blue reflectivity line, results of cold stimulations of the right and left ears; RE, right ear; LE, left ear; RN, right nystagmus; LN, left nystagmus.

Clinically, the irritative VES is identified based on three parameters: the head shaking test (HST), which triggers a nystagmus beating towards the pathological side; a kinetic test showing a preponderance towards the pathological side; and a caloric test showing an uncompensated deficit (Figure 2A). The deficient VES is identified with an HST triggering a nystagmus beating towards the healthy side, a kinetic test showing a preponderance towards the healthy side, and an uncompensated caloric test (Figure 2B).

##### 2.2.3.6 Study of subjective visual vertical and explanatory variables of its evolution

**Figure 3:**
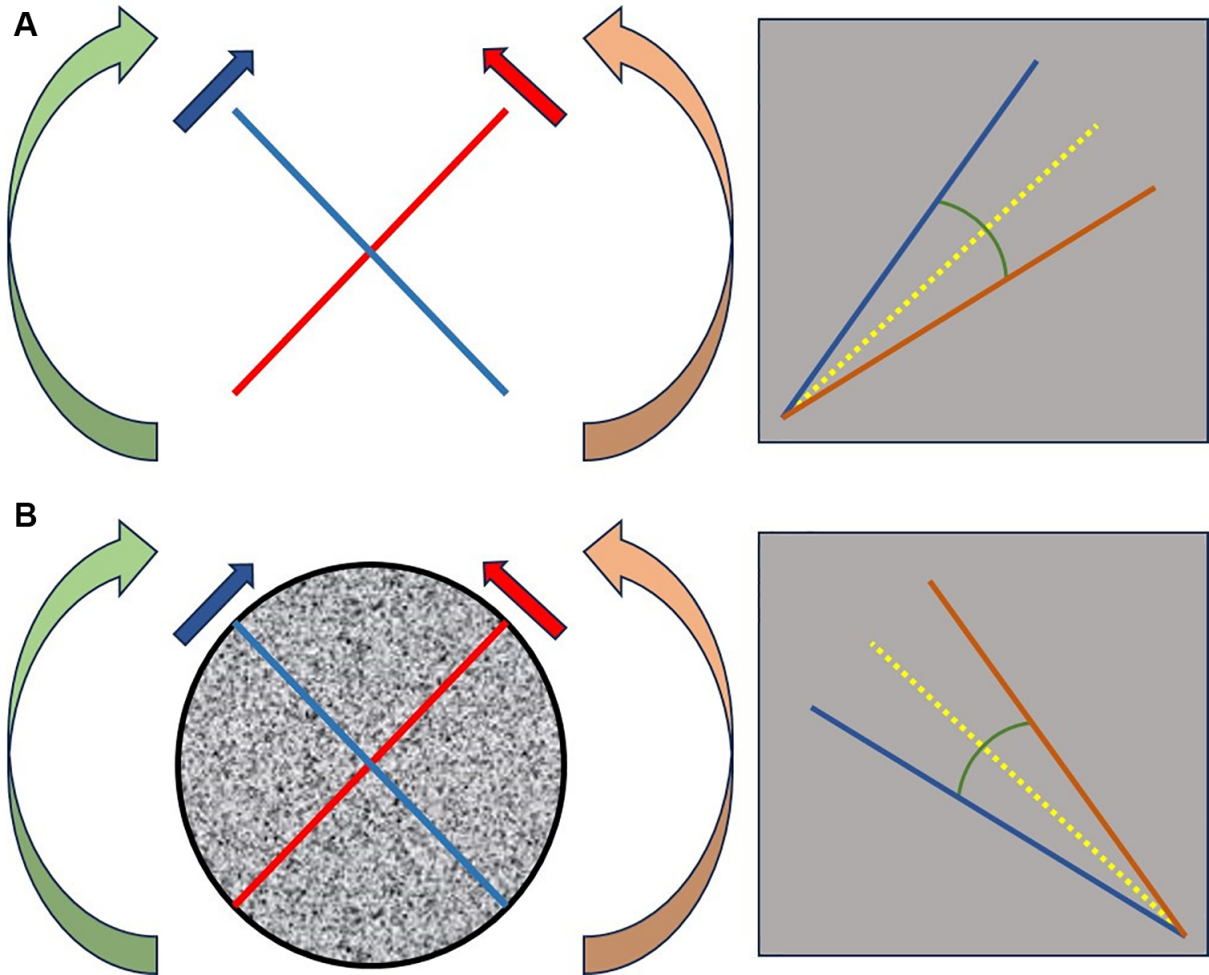
Description of the geometric angle and the bisector of the angle modeled on SVV measurements. We proceed with a random selection of the initial tilt. For example, for a right-sided selection: we perform a series of 4 measurements with the patient seated in darkness, starting from the right side [red line figure **(A)**], followed by 4 measurements from the left side [blue line figure **(A)**]. Each starting point is randomly positioned within an interval of [18°; 22°] on the right side and [−18°; −22°] on the left side relative to the vertical axis. Under dynamic conditions, optokinetic stimulation is initiated at 20°/s clockwise (green arrow) for measurements starting on the right [red line figure **(B)**], and counterclockwise (orange arrow) for measurements starting on the left [blue line figure **(B)**]. The same principles are applied except that we perform 6 measurements on each side. By averaging each series, we obtain 2 angles: one in static condition [figure **(A)**] and one in dynamic condition [figure **(B)**]. The bisector of each angle (yellow line) is then plotted. We evaluate the tracking of the geometric angle (closure = increased precision; opening = increased imprecision) and the variation of the bisector angle relative to the vertical (increased angle = decreased accuracy; decreased angle = increased accuracy).

We propose a new model of analysis for this work. The goal is to offer the community a new perspective on the examination and interpretation of the Subjective Visual Vertical (SVV; Figure 3) We chose to conduct four measurements on each side for the static test and six for the dynamic test because during our preliminary trials, we noticed that variations in measurements for certain VPC profiles, which are still poorly identified, either worsened or improved. This indicated the implementation of gravitational sensory-perceptive strategies, which we suspect are linked to somesthesia and graviception.

After averaging the measured values, we calculated for each static and dynamic condition a geometric angle (SGA and DGA, respectively) and a bisector for each angle obtained in static and dynamic conditions (SBA and DBA, respectively).

$$\theta \Delta =\;|\mu (\mathrm{SVVl}){\;}^{\circ }\text{-}\mu (\mathrm{SVVr}){\;}^{\circ }|$$

Where:

• *θΔ* = variation of the angle *θ*,
• *μ* = average of the angles in degrees (°) of the variables *SVVl* and *SVVr,*
• *SVVl*​ and SVVr = the set of measurements |to determine the angle SGA| taken on the right side (SVVstatr) and on the left side (SVVstatl); ­|to determine the angle DGA| taken on the right side (SVVdynr) and on the left side (SVVdynl), values expressed in degrees (°),
  $$\theta \Delta =\frac{\mu (\mathrm{SVVl}{)}^{\circ }+\mu (\mathrm{SVVr}){\;}^{\circ }}{2},$$

Where:

• *θΔ* = variation of the angle *θ,*
• *μ* = average of the angles taken by the bisector in the degree of inclination (°) for the values taken in SVVl and SVVr,
• SVVl and SVVr = the set of measurements |to determine the angle SBA| taken on the right side (SVVstatr) and on the left side (SVVstatl)/2 and |to determine the angle DBA| taken on the right side (SVVdynr) and on the left side (SVVdynl)/2, values expressed in degrees (°).

We modeled the geometric (Figure 3) angle obtained from the average amplitudes of the right and left test scores as representing precision (25). The bisector of the angle models accuracy. We hypothesize that accuracy is not solely linked to the internal model but also related to the integration of measurement error. In other words, two patients can have the same accuracy (represented by the inclination of the bisector relative to the vertical) but different opening angles (leading to different levels of precision: the more obtuse the angle, the lower the precision).

##### 2.2.3.7 Optometry test indicators from visual acuity measurements

We used two visual acuity measurement scales: the Monoyer scale (39) for distance visual acuity (DVA) assessment at 3 meters and the Parinaud scale for near visual acuity (NVA) assessment at 40 cm (40).

##### 2.2.3.8 Prismatic study (convergence and divergence) indicators

For accurate measurement of near convergence and divergence capabilities, we adopted the use of a prism bar (PB), combined with a specific measuring device (41). This device, consisting of a helmet equipped with a frontal axis on which a target is fixed at a distance of 30 cm from the nasion point, ensures uniform and reproducible measurements. The PB, with graduations extending from 1 to 40 diopters, is strategically positioned either base nasal for assessing divergence capabilities (PBd) or base temporal for examining convergence capabilities (PBc). Results are recorded in diopters.

##### 2.2.3.9 Optometry test indicators from the mawas board examinations

The Mawas Board, known as the Mawas-Weiss plate, consists of a cardboard plate with one side printed with a white line on a black background and the other side with a black line on a white background (42). We used this device to detect fusion disorders during vergence movements. Measurements were taken every 5 centimeters from 5 to 40 cm. A 10-s eyes-closed break was taken between each measurement to solicit a vergence movement from the rest position. Each measurement was taken randomly by drawing lots from 4 sequences for the initial assessment and 3 sequences for the final assessment (excluding the one obtained by lot during the first assessment). The goal was to closely mimic the ecological function of vergences. Fusion is considered normal when the subject visualizes a cross. Any other pattern is deemed abnormal.

##### 2.2.3.10 Optometry test indicators from measurements of near points of accommodation (NPA) and convergence (NPC)

We used an accommodation bar to measure positive NPAs (the distance at which maximum focus accommodation is achieved) and NPCs. The distance at which vision becomes blurry indicates the positive NPA in monocular use and the NPC in binocular use (43).

##### 2.2.3.11 Optometry test indicators from the Thomas Far stereoscopic vision test (TVST)

We assessed the patients' stereoscopic vision capabilities at distances of five and one meter, using four stereograms, based on the principle of Julesz's random dot stereograms (44). The first two, with a disparity of 250 arcs, featured images of a circle and a star, while the latter two, with a disparity of 300 arcs, depicted a cat and a car. These tests allowed measuring depth perception and the ability to distinguish spatial details at different distances.

##### 2.2.3.12 Optometry test indicators from the Worth four dot test

In our study, the Worth lamp was used as a diagnostic tool to assess patients' binocular perception. This instrument, consisting of a specific lighting system projecting four colored points (one red, two green, and one white) at different distances, helps detect binocular vision anomalies such as diplopia or suppression of one eye. The examination is considered normal when the colors generated by the 4 lamps are perceived in the following manner: i/red, ii/green, iii/green, iv/white or mixed color (42).

#### 2.2.4 Data preprocessing and univariate statistical analysis

The statistical analysis was conducted following an intention-to-treat strategy, where all participants were included in the analysis according to their initial allocation to the rehabilitation group. To handle missing values, we employed the mode imputation method, replacing missing values with the most frequently occurring category within our dataset, thus ensuring maximum data integrity. Data processing was performed to determine the evolution before (A1) and after (A2) rehabilitation with a threshold *p*-value of 0.05. The indicators of rehabilitation success are represented by the study of questionnaires. The search for tracking indicators is represented by the study of data from posturography, SVV, and optometry tests.

##### 2.2.4.1 Evaluation of responses to clinical questionnaires

The Shapiro-Wilk test assessed the normality of questionnaire scores before and after intervention, allowing the use of the Student's *t*-test or the Wilcoxon signed-rank test for comparing means, depending on the data distribution.

##### 2.2.4.2 Analysis of posturography indicators

We converted the continuous quantitative posturography scores into categorical variables, using normality thresholds defined by Synapsys. Values exceeding these thresholds were coded as “N” for normal and “AN” for abnormal. To examine the normality evolution between A1 and A2, we created four categories: “A” for variables abnormal at both A1 and A2, “B” for variables changing from abnormal to normal, “C” for those changing from normal to abnormal, and “D” for variables remaining normal. The frequencies of each category were calculated using a contingency table. The McNemar test was used to assess the statistical significance of variations.

##### 2.2.4.3 Analysis of kinetic VNG indicators

A statistical methodology was used to analyze the evolution of several indicators, including gains and preponderances at VVOR, VOR, OFI, VOR2, COR before (A1) and after (A2) rehabilitation. Data were categorized as “N” for normal and “AN” for abnormal according to specific thresholds. A frequency analysis documented the indicator evolution before and after rehabilitation. The McNemar test examined the significance of observed changes.

##### 2.2.4.4 Analysis of VOR2 and COR gain

A structured methodology was applied to analyze the evolution of VOR2 and COR gain, with classifications based on the improvement or deterioration of measurements. The Shapiro-Wilk tests, and depending on their results, Student's *t*-test or Wilcoxon signed-rank tests, evaluated significant differences.

##### 2.2.4.5 Comparative analysis of VOR and VOR2 gain trends

A comparative statistical analysis of VOR gain trends and VOR2 gain was used to determine their behavior between A1 and A2. For this, we created two continuous quantitative variables named:

• varVORg using VORgA1 and VORgA2 variables according to the following equation:
  $$\frac{\mathrm{VORgA}2\text{-}\mathrm{VORgA}1}{\mathrm{VORgA}1}\;\times 100$$

varVOR2g using VOR2gA1 and VOR2gA2 variables according to the following equation:

$$\frac{\mathrm{VOR}2\mathrm{gA}2\text{-}\mathrm{VOR}2\mathrm{gA}1}{\mathrm{VOR}2\mathrm{gA}1}\;\times 100$$

Sub-groups A and D from the VOR2 gain evolution study were used to create two new categorical variables (varVORgImprovement vs. varVOR2gImprovement and varVORgDeterioration vs. varVOR2gDeterioration) coding the VOR and VOR2 gains evolution between A1 and A2 into 3 categories: category 1 where VORg < VOR2g, category 2 where VORg and VOR2g observe a slight difference IC [−5.0; 5.0], and category 3 where VORg > VOR2g.

##### 2.2.4.6 Analysis of bithermal VNG reflectivity

To study the evolution of bithermal videonystagmography (VNGt) indicators between initial (A1) and final (A2) measurements, a two-phase statistical approach was adopted. Firstly, variations in these indicators were analyzed with statistical tests, classifying data by normality and using the McNemar test to evaluate changes in normality pre and post-rehabilitation. Secondly, evolution sub-groups (“A” for improvement, “D” for deterioration, and “I” for inversion of laterality) were formed. The Shapiro-Wilk test checked data normality, and differences were evaluated with the Wilcoxon signed-rank test. Comparisons between A1 and A2 measurements were performed to identify significant differences.

##### 2.2.4.7 Analysis of composite indicators: state of compensation (SoC) and hyperactive signal (Hs)

We descriptively identified different groups from these two classifications.

##### 2.2.4.8 Analysis of subjective visual vertical (SVV)

Subjects were classified according to the evolution of static (SGA) or dynamic (DGA) geometric angles between A1 and A2 into three categories: “D” for deterioration, “A” for improvement, and “S” for stagnation. This classification was also applied to the absolute values of bisector angles. If the absolute value of |static bisector angle (SBA) or dynamic (DBA) at A1| was strictly lower than |SBA or DBA at A2|, subjects were classified in the “D” category, if the absolute value of |SBA or DBA at A1| was strictly higher than |SBA or DBA at A2|, subjects were classified in the “A” category. The Shapiro-Wilk test checked the normality of distributions, with a threshold *p*-value of 0.05 to distinguish between normal and abnormal distributions. Comparisons of means between A1 and A2 for normally distributed variables were performed with the paired series Student's *t*-test, while the Wilcoxon signed-rank test was used for non-normally distributed distributions.

##### 2.2.4.9 Analysis of explanatory variables of SVV evolution

In this study, groups were defined based on the evolution of several key indicators: the cervical-ocular reflex (COR) gain, the state of compensation assessed by thermal videonystagmography (SoC), and the presence of a hyperactive signal (Hs). To analyze data variation and concentration, two statistical tools were used: the coefficient of variation (CV) and the Gini coefficient (Cg). The CV evaluates the dispersion of data around the mean, making the comparison between distributions with different means more equitable. A higher CV indicates a greater relative dispersion. The Cg measures data concentration, with values close to 0 indicating perfect equality and values close to 1, a high concentration. The combined use of CV and Cg allows assessing variability and concentration within groups, thus facilitating the comparison of homogeneity between them.

$$\mathrm{CV}=\left(\frac{\sigma }{m}\right)$$

Where:

• *σ* = standard deviation
• m = mean
  $$G=\frac{A}{A+B}$$

Where:

• G is a number between 0 and 1
• A represents the area between the Lorenz line and the line of perfect equality
• B represents the total area under the line of perfect equality.

##### 2.2.4.10 Analysis of optometry indicators

For the evolution of results obtained in the study of Near Visual Acuity (NVA), Distance Visual Acuity (DVA), prism convergence/divergence tests (PBc/PBd), Near Points of Accommodation (NPA), and Near Point of Convergence (NPC) between A1 and A2, averages were calculated. The distribution of data for normality was evaluated using the Shapiro-Wilk test. Statistical analysis of observed changes was performed using the Wilcoxon test for paired samples, suitable for non-parametric data.

The evolution of the normality state of measures in the Mawas Board examination, the Thomas Far Stereoscopic Vision Test (TVST), and the Worth test was studied, using the McNemar Chi-squared test to evaluate changes between A1 and A2.

#### 2.2.5 Data preprocessing and multivariate statistical analysis

We employed the Ordinary Least Squares (OLS) regression model to analyze the impact of selected variables on posturographic measurements and the SVV. The OLS model, with its equation

$$B=(X^{t}X{)}^{\text{-}1}(X^{t}Y)$$

aims to estimate the coefficients b, quantifying the influence of each independent variable X on the dependent variable Y. This method allows for the identification of causal relationships, unlike correlation analysis, which only detects co-variations. The statistical objective is to evaluate the impact of a set of explanatory factors on the variation of posturography data and the SVV angle between A1 and A2. The variation in posturography measurements was carried out according to the following model:

$$\Delta m=\frac{\mathrm{ma}1}{\mathrm{ma}2}\;\times 100-100$$

Where:

• Δm = variation of the measurement
• ma1 = measurement before iTRV
• ma2 = measurement after iTRV

For each evolution calculation, a quantitative variable was derived, on which linear regression was performed to measure the causality of potential explanatory factors. Predictive factors retained were all measured in the first period. Twelve variations in posturography measurements and four variations in angle measurements were thus calculated before a regression model was applied to each of them. In addition to the indicators to be explained, the study included a large set of potential explanatory variables. A selection process for these predictive factors was carried out in three steps.

First, for each variable to be explained, a univariate linear regression was performed for each potential explanatory variable. Variables from regressions with a *p*-value less than 25% were retained. Next, multicollinearity was examined to avoid selecting explanatory factors with a linear relationship that could explain the same variation. For this, the variance inflation factors (VIF) were calculated for each variable. Any variable with a VIF (adjusted for qualitative variables with more than two response modalities) greater than 5 was removed from the analysis. Finally, if necessary, a stepwise elimination procedure was carried out to retain only five exogenous variables. The final model retained was the one composed of five exogenous variables and presenting the lowest Akaike Information Criterion (AIC).

The quality of all models was evaluated by the coefficient of determination *R*^2^, which indicates the proportion of variance in the variable explained by the model's explanatory variables. The overall significance of the models was estimated by the Fisher test, where the null hypothesis assumes that none of the variables have a significant effect. The fit between the dependent variable and each independent variable was assessed by a Student's *t*-test, which tested the null hypothesis of no linear relationship between the dependent variable and the explanatory variable.

## 3 Results

### 3.1 Cohort presentation

**Figure 4:**
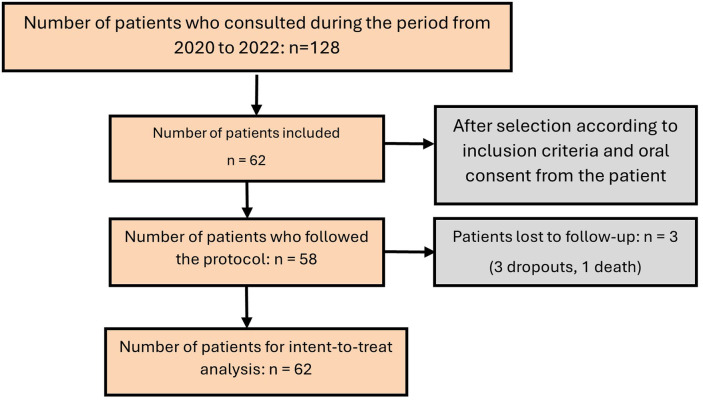
Flow chart.

A total of 62 patients were included (Figure 4). Our sample consisted of 45 women (72.6%) and 17 men (27.4%), with an average age of 59.4 years and a standard deviation of 18.1 years. The sample description is provided in Table 2.

**Table 2:** Study population characteristics (sample size 62).

Variables	Indicators
Year of Study Inclusion, *n* (%)	2021: 37 (59.7%), 2022: 25 (40.3%)
Follow-up Duration (months), Mean (SD)	13.0 (4.0)
Number of Sessions, Mean (SD)	86.6 (14.7)
Occupation, *n* (%)	Business Owner: 1 (1.6%), Freelance Professional: 1 (1.6%), Executive or Higher Intellectual Profession: 3 (4.8%), Intermediate Profession: 10 (16.1%), Employee: 11 (17.7%), Worker: 2 (3.2%), Retired: 29 (46.8%), Homemaker: 4 (6.5%), Student: 1 (1.6%)
Engagement in Sports Activity, *n* (%)	26 (41.9%)
Initial Diagnosis, *n* (%)	Other initial conditions: 26 (41.9%), Chronic Unilateral Vestibular Hypofunction (CUVH): 6 (9.7%), Undefined: 9 (14.5%), Recurrent Benign Paroxysmal Positional Vertigo (rBPPV): 21 (33.9%)
Diagnosis at Inclusion (A1), *n* (%)	Other: 25 (40.3%), CUVH: 16 (25.8%), Undefined: 15 (24.2%), rBPPV: 6 (9.7%)
Diagnosis at End of Care (A2), *n* (%)	Other final conditions: 28 (45.2%), CUVH: 5 (8.1%), Persistent Postural-Perceptual Dizziness (PPPD): 11 (17.7%), Functional (Psychogenic) Vertigo: 17 (27.4%), rBPPV: 1 (1.6%)

“Other” in initial diagnosis includes unilateral vestibular schwannoma, Ménière's disease. “Other” in final diagnosis includes unilateral vestibular schwannoma, Ménière's disease, vestibular migraine, Friedrich's disease; CUVH, chronic unilateral vestibular hypofunction; PPPD, persistent postural-perceptual dizziness; Functional (Psychogenic) Vertigo, psychiatric diagnosis made after the start of rehabilitative care: phobic disorders, bipolar affective disorder, anxiety disorder, major depressive disorder, post-traumatic stress disorder, and somatoform disorder; rBPPV, recurrent benign paroxysmal positional vertigo. Care: management.

The patients lost to follow-up represented 6.5% of the cohort. Among these patients, the diagnosis evolved after the start of the rehabilitative intervention: two for Canvas, five and nine months later, one for Friedrich's ataxia six months later, and one due to suicide 10 months after starting the rehabilitative follow-up. Two diagnoses of vestibular migraine were reevaluated seven months and one year later. The initial diagnosis of recurent Benign Paroxysmal Positional Vertigo (rBPPV) accounted for 33.9% but was reduced to 1.6% by the end of rehabilitation. 24.2% of undefined vestibular vertigos were defined by the end of care.

During the first crisis, 51.6% of the cohort reported experiencing rotational type visual vertigo, triggered by movement in 77.4% of cases and transient in 54.8% of cases; triggered by vision in 38.7% of cases, by Valsalva maneuver in 9.7% of cases, and by orthostatism in 9.7% of cases. Blurred vision induced by movement at the first crisis was present in 11.3% of the cohort and increased to 59.7% of the cohort at the first physiotherapy consultation. Other visual symptoms identified during the interview are presented in Table 3.

**Table 3:** Visual symptoms reported at the first physiotherapy consultation (sample size 62).

Variables	Indicators, *n* (%)
Change observed by patient since first Episode	56 (90.3%)
Fatigue when reading	24 (38.7%)
Wearing progressive glasses	21 (33.9%)
Oscillopsia	3 (4.8%)
Intermittent diplopia	7 (11.3%)
Movement-induced blurry vision	37 (59.7%)
Decrease in near visual acuity (NVA)	46 (74.2%)
Decrease in visual field while driving	32 (51.6%)
Other Symptoms	Photophobia: 6 (9.8%), Visual Vertigo While Watching TV: 17 (27.9%)

NVA, near visual acuity.

Regarding general health, 40.3% of the cohort experienced sleep disorders, and 91.9% reported abnormal fatigue that gradually set in after the first crisis. Notably, before the first crisis (one year aflter): 72.6% of the cohort had anxiety disorders, among them: 27.4% had at least one depressive episode, and 30.5% were followed for post-traumatic stress disorder. 1.61% of the cohort suffered from Temporomandibular Joint Disorders (TMJD) before the first crisis compared to 14.5% at the first consultation; 1.61% had facial nerve damage compared to 9.68%, and 3.13% had chronic neck pain compared to 25.8%, which is a quarter of the cohort.

### 3.2 Evaluation of iTRV: questionnaire analysis

**Figure 5:**
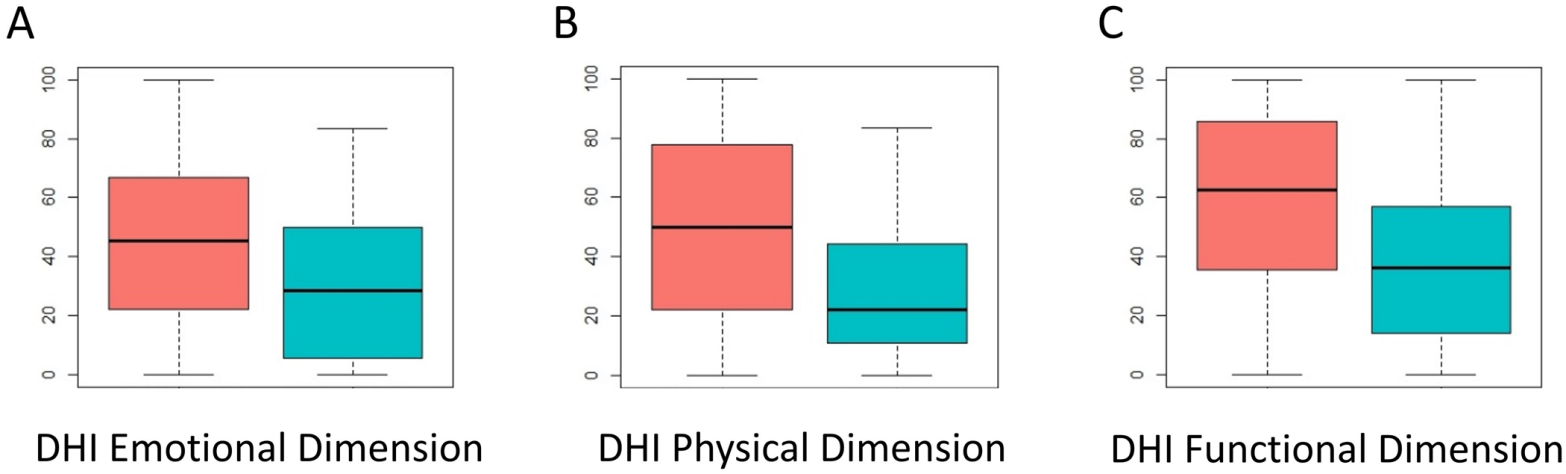
Distribution of scores across the three components of the dizziness handicap inventory (DHI). Red: scores at A1; Blue: scores at A2. Higher scores indicate a poorer state of the evaluated component.

**Figure 6:**
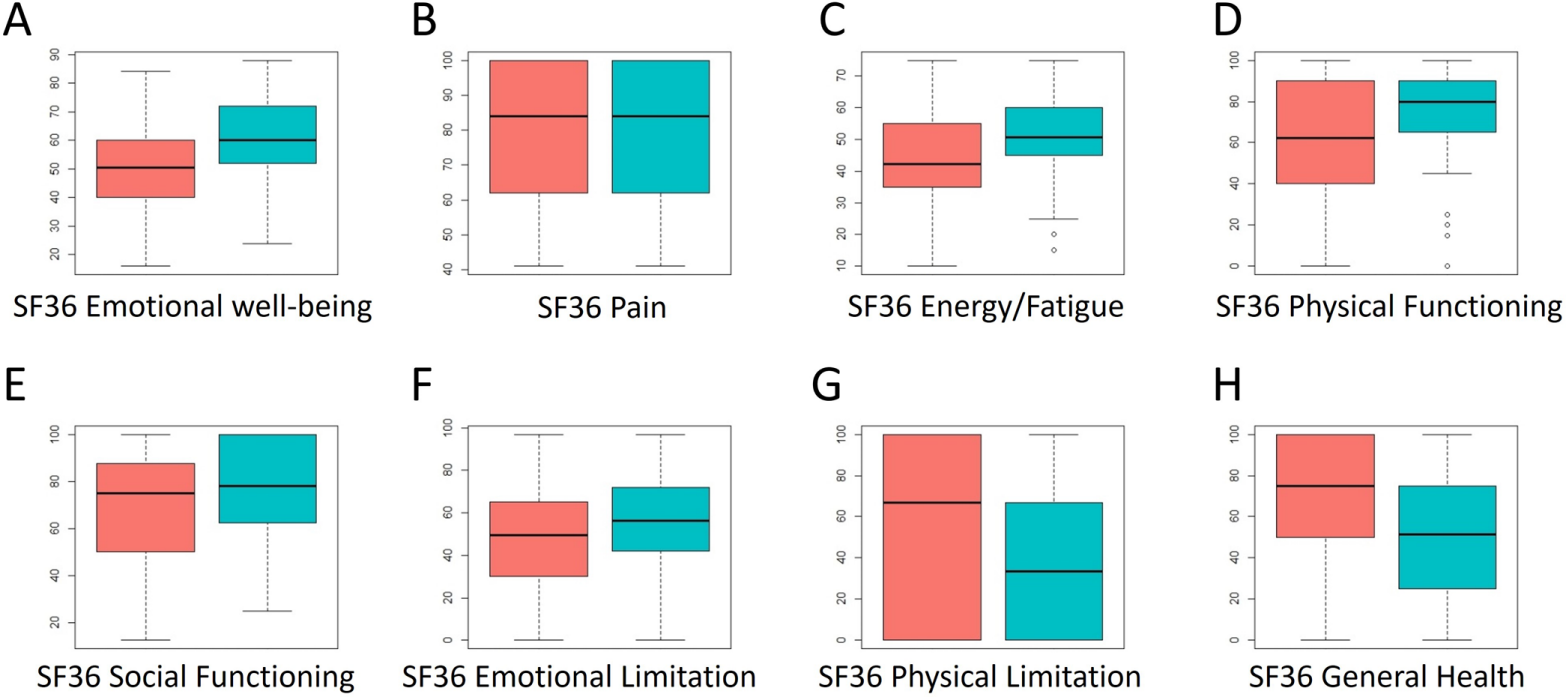
Distribution of scores across the eight dimensions of the SF36 questionnaire. Red: scores at A1; Blue: scores at A2. Higher scores indicate a better state of the evaluated component.

**Figure 7:**
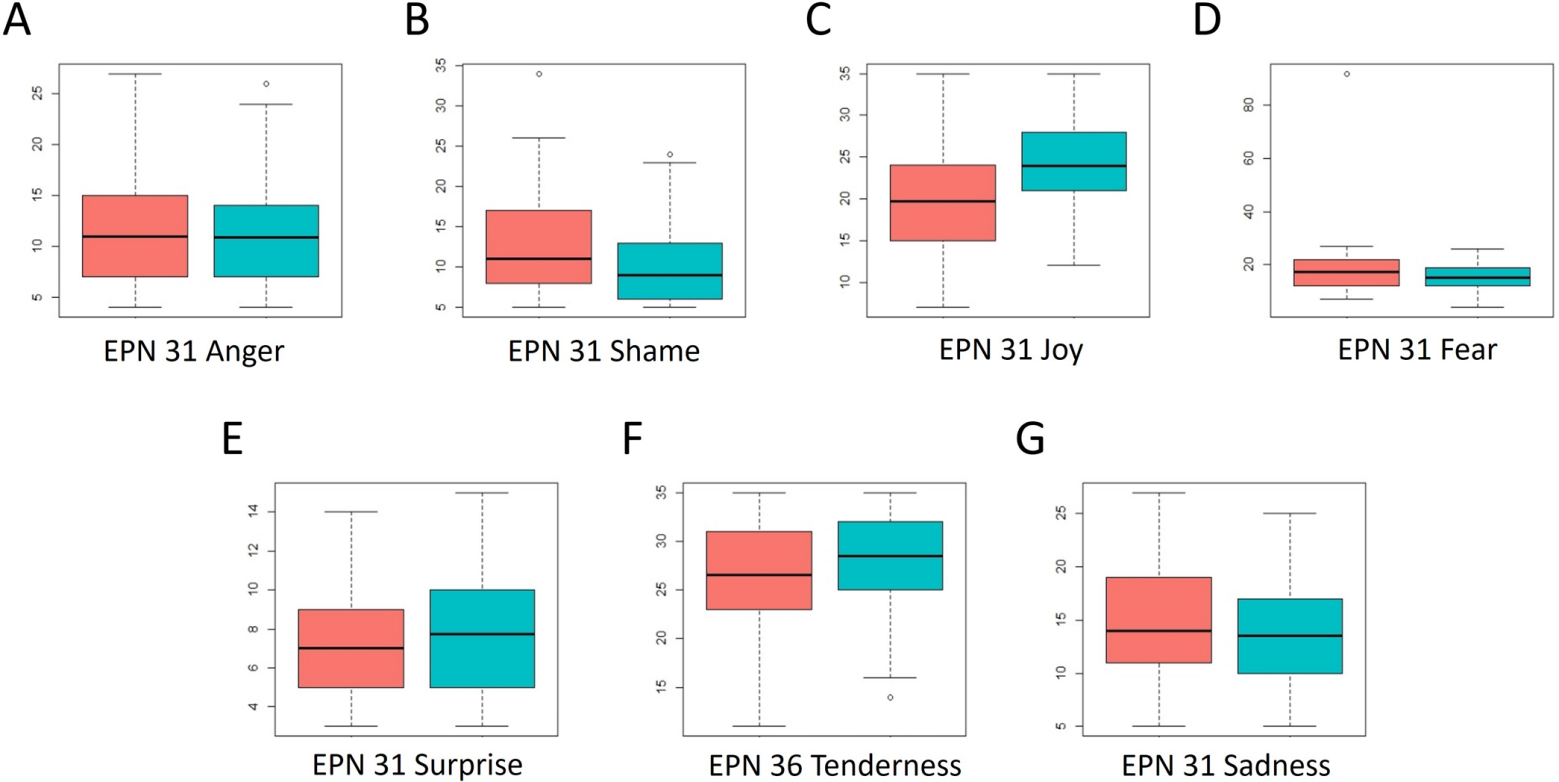
Distribution of scores across the five dimensions of the EPN-31 questionnaire. Red: scores at A1; Blue: scores at A2. Higher scores indicate that the evaluated emotional component is experienced more frequently, and vice versa.

**Figure 8:**
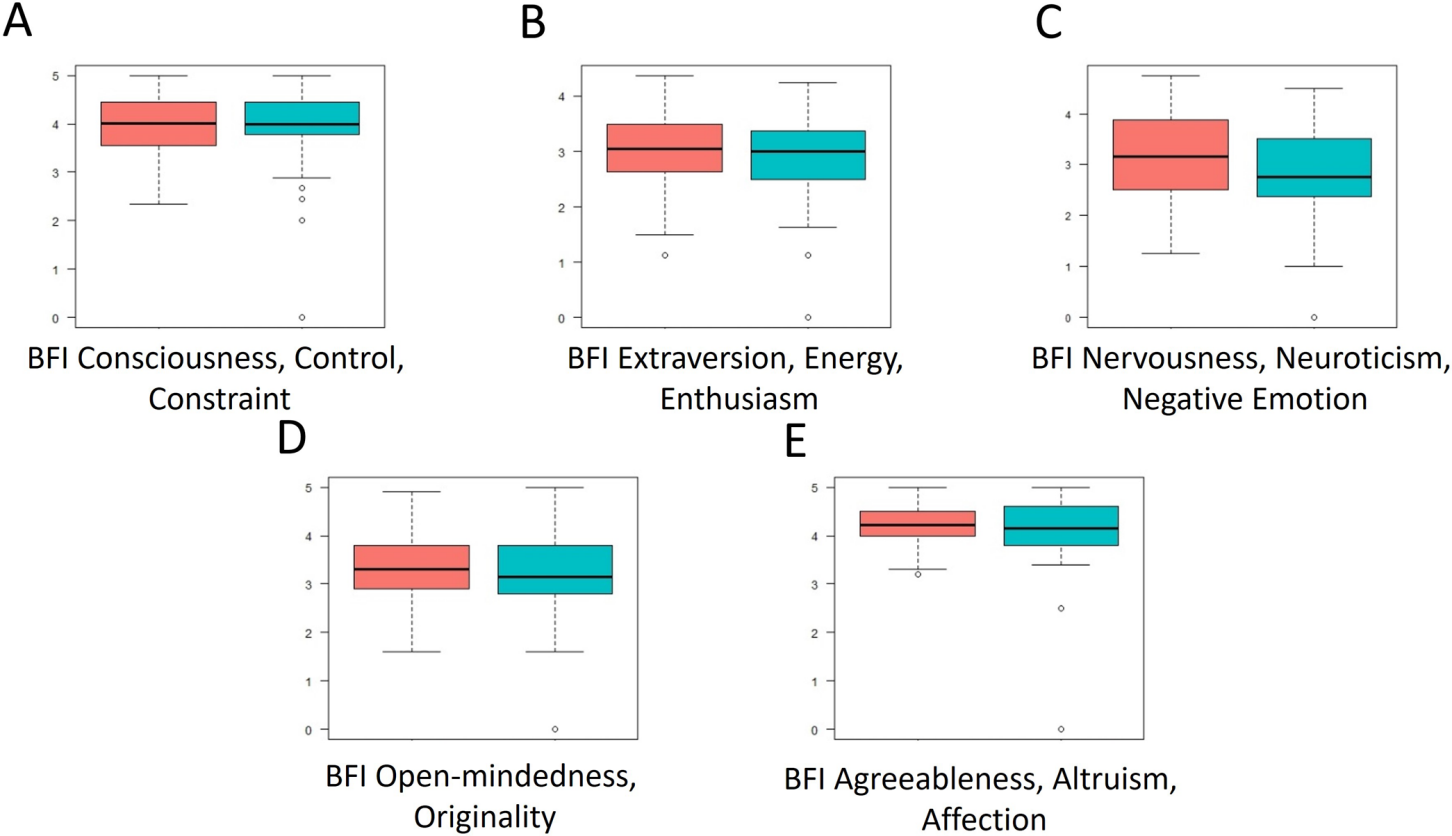
Distribution of scores across the five dimensions of the BFI questionnaire. Red: scores at A1; Blue: scores at A2. The higher the score, the more pronounced the corresponding personality trait (Extraversion, Agreeableness, Conscientiousness, Neuroticism, Openness to Experience), and vice versa.

**Figure 9:**
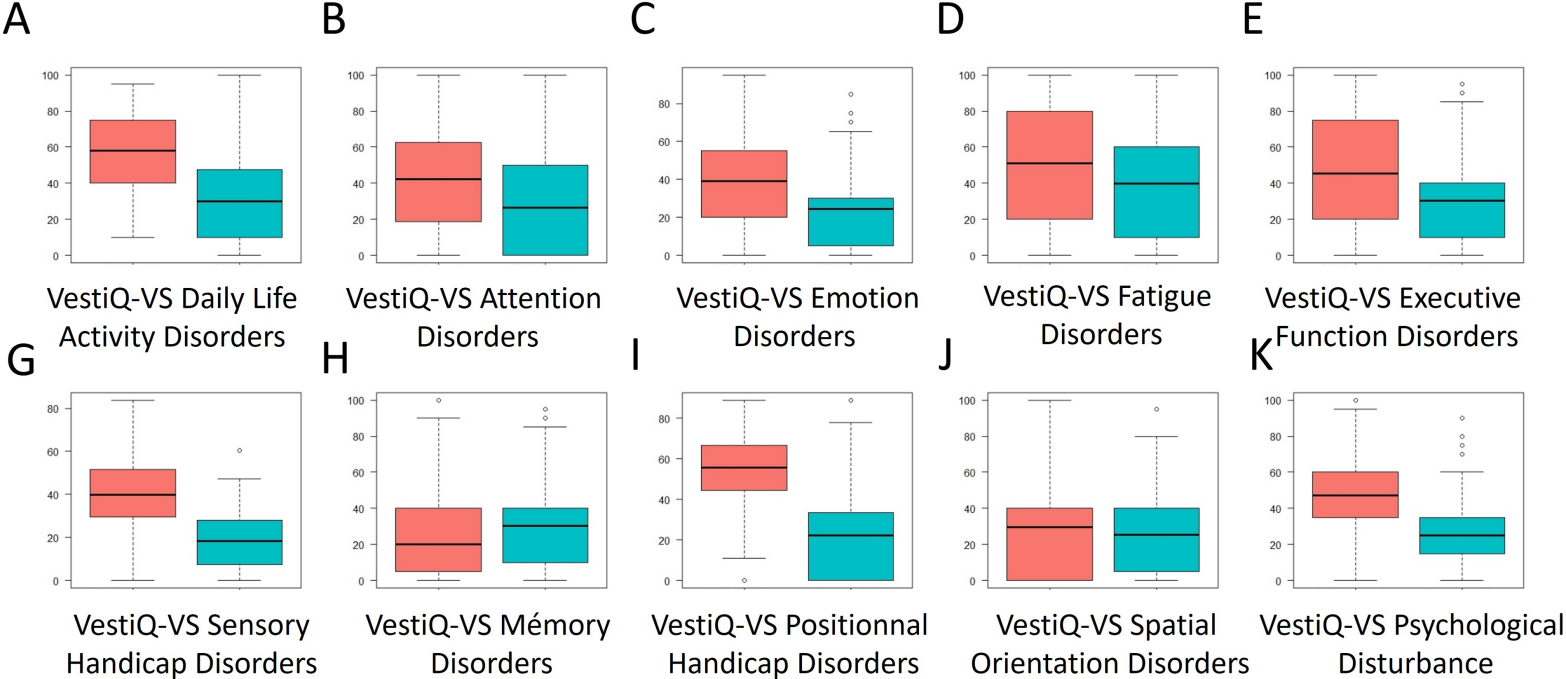
Distribution of scores across the ten dimensions of the vestiQ-VS questionnaire. Red: scores at A1; Blue: scores at A2. The higher the score, the more deteriorated the state of the evaluated component.

All results are presented in Figures 5–9, and all statistical results in Supplementary Table S9 due to the data density. In an unconventional manner to facilitate data approach, we present a list from the analysis of score variation that is not significant (*p* > 0.05) for the following dimensions: SF36 pain, EPN anger, EPN surprise, BFI Extraversion, Energy, Enthusiasm, BFI Agreeableness, Altruism, Affection, BFI Conscientiousness, Control, Constraint, BFI Openness, Originality, Open-mindedness, VestiQ-VS memory, and VestiQ-VS spatial orientation.

At the end of integrative vestibular rehabilitation therapy (iVRT): 79% of patients presenting abnormal fatigue improved their scores in the fatigue dimension of the VestiQ-VS questionnaire, 78.72% of patients who presented anxiety disorders improved their emotion management score (EPN 31 questionnaire) and reported having improved their anxiety state either by decreasing medication or by resuming activities that had become anxiety-inducing before rehabilitation. Finally, 75% of patients suffering from neck pain improved their score in the pain dimension of the SF36.

### 3.3 Analysis of instrumental tracking indicators

#### 3.3.1 Posturography indicator analysis

The statistical study of the variation in BOS scores gives us significant results for the evolution of i/ Vestibular score in mediolateral imbalance condition: McNemar's chi-squared = 4.00, dF = 1, *p*-value = 0.046; ii/ Composite score in mediolateral imbalance condition: McNemar's chi-squared = 6.13, dF = 1, *p*-value = 0.01.

#### 3.3.2 Analysis of indicators from kinetic VNG tests

To assess whether rehabilitation impacted the VVOR, VOR, VOR2, COR, and OFI indicators, we examined the evolution of normality (transition to norms or not) of these indicators between two points in time: before (A1) and after (A2) for preponderance and gain. No results were statistically significant.

#### 3.3.3 Analysis of VOR2 gain (VOR2g) and COR gain (CORg)

Three subgroups were created for the analysis of VOR2 gain as a continuous quantitative variable to assess the variation of VOR2 gain between A1 and A2. Group A: increase *n* = 22, D: decrease *n* = 36, S: stability *n* = 0.

• For subgroup A: V = 0, *p*-value = 1.93e-05 shows a significant difference between the VOR2g means at A1 and A2 in this subgroup. The mean differences (signed Wilcoxon test) suggest a significant improvement in VOR2g mean after rehabilitation for subjects in subgroup A.
• For subgroup D: t = 8.46, dF = 37, *p*-value = 3.59e-10 also shows a significant difference between the VOR2g means at A1 and A2 in this subgroup. The mean differences suggest a significant deterioration in VOR2g mean after rehabilitation.

Three subgroups were created for the analysis of numerical COR gain as a continuous quantitative variable to assess the variation of COR gain between A1 and A2. Group A: increase *n* = 25, D: decrease *n* = 31, S: stability *n* = 5.

• For subgroup A (improvement): V = 496, *p*-value = 1.22e-06. A significant difference between the CORg means at A1 and A2 for subjects classified as A is demonstrated (signed Wilcoxon test). This suggests a significant improvement in CORg after rehabilitation.
• For subgroup D (deterioration): V = 0, *p*-value = 8.752e-06 also shows a significant difference between the CORg means at A1 and A2 for subjects classified as D (signed Wilcoxon test). This suggests a significant deterioration in CORg after rehabilitation.

#### 3.3.4 Comparative analysis of VOR (VORg) and VOR2 (VOR2g) gain trends

Among the patients with a statistically significant variation in VOR2 gain (*n* = 43), two evolution groups were observed: group A: group observing an increase in VOR2 gain, and group D: group observing a decrease in VOR2 gain.

In each group, 3 behaviors were identified:

• Group A *n* = 24: condition 1 (VORg < VOR2g) *n* = 14, condition 2 *n* = 2: VORg and VOR2g observe a slight difference IC [−5, 5], condition 3 *n* = 8: VORg > VOR2g
• Group D *n* = 19: condition 1 (VORg < VOR2g) *n* = 12, condition 2 *n* = 5: VORg and VOR2g observe a slight difference IC [−5, 5], condition 3 *n* = 2: VORg > VOR2g

#### 3.3.5 Analysis of reflectivity from the bithermal VNG test

To study the significance of the evolution of this indicator according to its clinical interpretation, three subgroups were created: subgroup A where reflectivity improved after rehabilitation, subgroup D for which reflectivity deteriorated after rehabilitation, subgroup I where reflectivity reversed its laterality after rehabilitation.

For subgroup A and D, we compared pairs of values measured at A1 and A2 to see if the position of the medians is different from 0. This test, being conducted by pairs of values on the same variable measured at two moments, it is impossible to compare the evolution of group I, as the change in the laterality of reflectivity does not allow the statistical test to be applied.

• For subgroup A: the evaluation of right-side reflectivity gives a V = 0, *p*-value = 0.016, the evaluation of left-side reflectivity gives a V = 0, *p*-value < 0.001.
• For subgroup D: the evaluation of right-side reflectivity gives a V = 36, *p*-value = 0.008, the evaluation of left-side reflectivity gives a V = 36, *p*-value = 0.008.

These results suggest that, for each pair of variables and for each subgroup, there is a significant difference between the two variables. The alternative hypotheses indicate that the true difference in position is not equal to zero, meaning that the medians of the two groups are different.

#### 3.3.6 Analysis of composite indicators

11.29% present a hyperactive signal (Hs) at the beginning of rehabilitation (A1), that is 7 patients, 0% at the end of care (A2).

The state of compensation (SoC) in our cohort is distributed as follows: at A1, 46.6% have normal reflectivity with a deficit ≤ 30%, 36.6% have reflectivity ≤ 15¨/s with a deficit ≥ 30%, 1.7% present bilateral areflexia (reflectivity ≤ 2¨/s with a deficit ≥ 70%), and 15% unilateral hypovalence without deficit (reflectivity ≤ 15¨/s with a deficit ≤ 30%). At A2, the proportions are 46.6% with normal reflectivity, 18.3% with reflectivity ≤ 15¨/s and deficit ≥ 30%, 6.7% with bilateral areflexia, and 6.7% with unilateral hypovalence without deficit. Between A1 and A2, 18 patients changed their SoC during rehabilitation, against 42 who did not change.

#### 3.3.7 Evolution of geometric angles and bisectors

Three subgroups were created according to the conditions of improvement (A) or deterioration (D) of the SVV between A1 and A2. The study of the normality of variables from the analysis of the SVV with the Shapiro-Wilk test is available in the Supplementary Table S10. For each group, the following distribution is observed:

• Group A: SGA *n* = 36, SBA *n* = 42, DGA *n* = 32, DBA *n* = 41
• Group D: SGA *n* = 26, SBA *n* = 20, DGA *n* = 30, DBA *n* = 31

The statistical study of variations for each group gives the results described in Table 4.

**Table 4:** Statistical results of SVV measurement variations by group.

Population	Variable	Wilcoxon statistic	*p*-value
SGA A	Static Geometric Angle A1 vs. A2	666	<0.001***
SBA A	Static Bisector Angle A1 vs. A2	407	0.58
DGA A	Dynamic Geometric Angle A1 vs. A2	528	<0.001***
DBA A	Dynamic Bisector Angle A1 vs. A2	346	0.28
SGA D	Static Geometric Angle A1 vs. A2	−6.87	<0.001***
SBA D	Static Bisector Angle A1 vs. A2	−1.02	0.32
DGA D	Dynamic Geometric Angle A1 vs. A2	−6.10	<0.001***
DBA D	Dynamic Bisector Angle A1 vs. A2	0.66	0.52

SGA, static geometric angle; SBA, static bisector angle; DGA, dynamic geometric angle; DBA, dynamic bisector angle; A, Improvement group,;D, deterioration group.

* trend towards significance.

** moderate significance.

*** strong significance.

A descriptive statistical analysis of the variation of SVV indicators (SGA, DGA, SBA, DBA) by the coefficient of variation (CV) and the Gini coefficient (Cg) was performed based on the grouping factors identified a posteriori (presence or absence of a hyperactive signal (Hs), compensation profiles either stable or evolved during iVRT (SoC) and improvement/decrease of the gain obtained at the cervical-ocular reflex(CORg). The results are presented in Tables 5, 6. The evolution of SVV measurements between A1 and A2 is available in the Supplementary Table S11.

**Table 5:** Homogeneity of SVV variation by post-Hoc group formation at A1.

Variable	Group	Sample size	Static SVV geometric angle CV (Cg)	Static SVV bisector angle CV (Cg)	Dynamic SVV geometric angle CV (Cg)	Dynamic SVV bisector angle CV (Cg)
Hyperactive signal	Absent	55	0.5 (0.27)†	0.8 (0.41)	0.5 (0.26)†	1.1 (0.5)
	Present	7	0.9 (0.39)	0.8 (0.39)	0.7 (0.38)	0.4 (0.22)†
State of compensation	Constant	42	0.6 (0.29)	0.8 (0.44)	0.5 (0.29)	1.1 (0.49)
	Variable	18	0.5 (0.28)†	0.8 (0.39)	0.4 (0.21)†	1.0 (0.5)
COR gain	Increase	25	0.5 (0.26)	0.8 (0.41)	0.4 (0.24)	1.3 (0.57)
	Stable	5	0.4 (0.17)†	0.7 (0.31)	0.4 (0.19)†	1.8 (0.67)
	Decrease	31	0.7 (0.32)	0.8 (0.44)	0.6 (0.3)	0.8 (0.43)

The groups with the most homogeneity in measurement are indicated by † CV represents the coefficient of variation, and Cg stands for the Gini coefficient, both assessing the dispersion and equality of SVV variations among the groups.

**Table 6:** Homogeneity of SVV variations by post-Hoc group formation at A2.

Variable	Group	Sample size	Static SVV geometric angle CV (Cg)	Static SVV bisector angle CV (Cg)	Dynamic SVV geometric angle CV (Cg)	Dynamic SVV bisector angle CV (Cg)
Hyperactive signal	Absent	55	0.5 (0.27)†	10,9 (0,42)	0.5 (0.26)†	4,9 (0,50)
	Present	7	0.4 (0.39)		4.3 (0.44)	
State of compensation	Constant	42	0.5 (0.29)†	4,3 (0,44)	0.5 (0.29)	24,9 (0,41)
	Variable	18	0.6 (0.28)		0.5 (0.21)†	
COR gain	Increase	25	0.4 (0.28)†		0.5 (0.26)†	
	Stable	5	0.6 (0.17)		0.6 (0.186)	
	Decrease	31	0.6 (0.32)	7 (0,44)	0.6 (0.3)	31,8 (0,43)

Groups with the most homogeneous measures are indicated by †. CV denotes the coefficient of variation, and Cg is the Gini coefficient, both used to measure the dispersion and equality of SVV variations among the groups. The columns for static and dynamic SVV bisector angles are omitted due to negative values (improvement towards 0 indicates less deviation) and the challenges in interpreting CV and Cg for these measures.

#### 3.3.8 Analysis of results obtained by optometry indicators

##### 3.3.8.1 Results from the analysis of visual acuity

The evolution of Near Visual Acuity (NVA; Table 7) shows a statistically significant improvement (*p* < 0.001).

**Table 7:** Visual acuity variation study from baseline (A1) to follow-Up (A2).

Data	Mean at A1	Mean at A2	Increase proportion	Decrease proportion	No change proportion	Average rate of change	*P*-value
Distance visual acuity (DVA)	8.66	7.39	4.9%	11.3%	83.8%	−4.0%	0.334
Near visual acuity (NVA)	2.83	2.4	3.3%	44.3%	52.5%	−13.0%	<0.01***

* Trend towards statistical significance (*P*-value > 0.05).

** Moderate statistical significance (0.01 < *P*-value ≤ 0.05).

*** Strong statistical significance (*P*-value ≤ 0.01).

**Reading Key:** This table presents the changes in both distance and near visual acuity from the initial assessment (A1) to the follow-up assessment (A2), highlighting the proportions of individuals experiencing increases, decreases, or no change in visual acuity, alongside the average rate of change and their statistical significance.

##### 3.3.8.2 Results from prismatic analysis (PBc/PBd)

The evolution of convergence and divergence capabilities at the prism bar (PBc/PBd; Table 8) is not statistically significant.

**Table 8:** Study of variations in convergence and divergence (PBc/PBd) from baseline (A1) to follow-Up (A2).

Data	Number at A1	Number at A2	Mean at A1	Mean at A2	Increase proportion	Decrease proportion	Average rate of change	*P*-value
Nasal right eye (OD)	61	60	11.51	11.8	47%	32%	11%	0.561
Nasal left eye (OG)	60	60	10.98	11.43	47%	38%	14%	0.678
Temporal right eye (OD)	43	52	22.95	23.17	35%	48%	13%	0.851
Temporal left eye (OG)	44	52	23.16	23.46	33%	56%	20%	0.771

PBc, prism bar convergence; PBd, prism bar divergence. **Reading Key:** Variations were not calculated for patients who had neutralization at baseline (A1) or follow-up (A2). The average rate of change is not the change in mean values from A2 compared to A1 but is the average rate of change for each patient. This table outlines the variations in convergence and divergence capabilities, as measured by the prism bar, from the initial assessment to the follow-up assessment. It includes details on the average measures at each time point, the proportion of individuals who saw increases or decreases in capabilities, and the overall average rate of change across the study population.

##### 3.3.8.3 Results from the analysis obtained at the mawas board (PmW)

The analysis of the variation in measurements obtained during the PmW examination is presented in Figure 7. A McNemar's Chi-squared test was applied to determine if the discordant pairs evolved through rehabilitative intervention:

• PmW20 A1A2 McNemar's chi-squared = 6.86, dF = 1, *p*-value = 0.01***

All results is available in the Supplementary Material section, Table S12.

##### 3.3.8.4 Results from the analysis of measurements of near points of accommodation (NPA) and near point of convergence (NPC)

To analyze the evolution of NPA right, NPA left, and NPC values between A1 and A2, the variation in means between these two periods was examined.

• For the improvement subgroup (A) of NPA right, NPA left, and NPC values between A1 and A2, the signed Wilcoxon test shows that the differences are significant with very low *p*-values, indicating significant improvements.
• For the deterioration subgroup (D), the signed Wilcoxon test also shows significant differences with very low *p*-values, indicating significant deteriorations.

##### 3.3.8.5 Results from the analysis of the Thomas Far stereoscopic vision test (TVST)

The analysis of the variation in measurements obtained during the far stereoscopy (TVST) exam evaluated by four figures (circle, star, cat, car) is presented in Figure 9. A McNemar's Chi-squared test was applied to determine if the discordant pairs evolved through rehabilitative intervention:

• Star 1 m A1A2: McNemar's chi-squared = 5.26, dF = 1, *p*-value = 0.02**
• Car 1 m A1A2: McNemar's chi-squared = 5.33, dF = 1, *p*-value = 0.02**
• Circle 5 m A1A2: McNemar's chi-squared = 5.06, dF = 1, *p*-value = 0.02**
• Star 5 m A1A2: McNemar's chi-squared = 4.08, dF = 1, *p*-value = 0.04**

All results is available in the Supplementary Material section, Table S13.

##### 3.3.8.6 Results from the analysis of the Worth test

The analysis of the variation in measurements obtained during the Worth four dot test (Table 9) shows a statistically significant change between A1 and A2 (*p* < 0.01), indicating how the perception of color and binocular vision may have changed following rehabilitation. For 26.7% of patients, we observe a restoration of retinal correspondence, and for 8.3%, an alteration of retinal correspondence (*p* = 0.029).

**Table 9:** Study of variations in convergence and divergence (PBc/PBd) measures from baseline (A1) to follow-Up (A2).

Data	Red point	Green point	Green point	White point
Number of changes from A1 to A2	0	1	0	46
Frequency of changes from A1 to A2	0.0%	1.7%	0.0%	76.7%
Frequency of accurate tests at A1	0.0%	0.0%	0.0%	13.1%
Frequency of accurate tests at A2	0.0%	0.0%	0.0%	31.1%
*P*-value 1	>0.99	>0.99	>0.99	<0.01***
Maintained norms from A1 to A2	0.0%	0.0%	0.0%	5.0%
Norms at A1, not at norms at A2	0.0%	0.0%	0.0%	8.3%
Not at norms at A1, at norms at A2	0.0%	0.0%	0.0%	26.7%
Not at norms at A1 and A2	100.0%	100.0%	100.0%	60.0%
*P*-value 2	>0.99	>0.99	>0.99	0.029**

*P*-value 1 evaluates whether patients changed their response (regardless of the response's correctness). *P*-value 2 compares changes in response status between the two periods, grouping incorrect responses (all but white, yellow, and orange) to assess changes from good to non-good. PBc, prism bar convergence; PBd, prism bar divergence.

* Trend towards statistical significance (*P*-value > 0.05).

** Moderate statistical significance (0.01 < *P*-value ≤ 0.05).

*** Strong statistical significance (*P*-value ≤ 0.01).

**Reading Key**: This table presents the variations in responses to convergence and divergence tests, highlighting the significant changes observed between the baseline and follow-up evaluations. It details the proportion of patients experiencing changes and assesses the accuracy of tests over time, providing a clear view of the shifts in visual function related to these specific tasks.

### 3.4 Search for predictive markers

#### 3.4.1 Presentation of results

This section presents significant results. Twelve conditions were treated representing the six trials of the Sensory Organization Test (SOT) with each trial, the conditions of anteroposterior (AP) and mediolateral (ML) imbalance. Four models to explain posturography were retained; we did not use a method to adjust the significance threshold since our models did not include the same regressors. In addition to the significance of a factor's effect on the endogenous variable, the models allowed us to determine the explanatory power of each explanatory variable through the regression coefficients (*β*). Finally, one last model was retained concerning the evolution of the SVV bisector angle, but in a categorical form. The explained variable took the “improvement” modality if the angle in the second measurement approached 0 degrees, the “deterioration” modality otherwise. The objective was to evaluate the impact on the direction of the SVV bisector angle variation of the five explanatory variables: the ML-assisted posturo-static, the ML-assisted posturo-dynamic, the COR gain, the VOR preponderance, and whether the Romberg quotient (QR) was within norms or not.

To measure these potential cause-and-effect relationships, a multivariate and multinomial logistic regression was performed. The model's adjustment was determined by calculating McFadden's pseudo-R^2^, the significance of the co-factors' impact by ANOVA, and the results expressed as odds ratios.

#### 3.4.2 Evolution of medio-lateral balance

The regression of the variation of the total energy (E) measurement in static (St) condition with eyes open (EO) for ML balance is significant (*P* < 0.01) and accounts for 31% of the variance. The model shows a causality of the dimension dBIG5A and the SoC component T at the 5% threshold. All results are presented in Table 10.

**Table 10:** OLS regression analysis ΔEStEOML.

Variables	Beta Coefficient	Confidence Intervals	*P*-value
Constant	−264	[−546; 18]	0.073*
**dSE** (emotion dimension VestiQ-VS)	−4	[−9.2; 1.1]	0.123
**dSF36SG** (general health dimension SF36)	1	[−0.20; 2.3]	0.1*
**dEPN31P** (fear dimension EPN31)	−0.85	[−2.9; 1.2]	0.417
**dBIG5A** (Agreeableness, Altruism, Affection)	7.6	[1.5; 14]	0.014**
**SoC**			0.132
N			
I	70	[−24; 165]	0.151
P	23	[−30; 75]	0.402
T	98	[−4.2; 191]	0.047**
Model global statistics	R^2^: 0.311	Fisher statistics: 3.377	Fisher test (*p*-value): 0.012**

* Trend towards statistical significance (*P*-value > 0.05).

** Moderate statistical significance (0.01 < *P*-value ≤ 0.05).

*** Strong statistical significance (*P*-value ≤ 0.01).

**Reading key:** *Δ*EStEOML = *β*(0)+*β*1 × dSE+*β*2 × dSF36SG+*β*3 × dEPN31P+*β*4 × dBIG5A+*β*5 × EdCI+*β*6 × EdCN+*β*7 × EdCP+*β*8 ×  EdCT. dSE = Emotional dimension of the VestiQ-VS questionnaire, dSF36SG = General health dimension of the SF36 questionnaire, dEPN31*P* = Fear dimension of the EPN31 questionnaire, dBIG5A = Agreeableness, Altruism, Affection dimension. State of Compensation (SoC) classifications: (N) Non-Inhibited Profile with ≥15°/s contralateral reflectivity and ≤30% ipsilateral deficit, showing no subcortical arc modulation; (P) Partial Contralateral Inhibition Profile, with [2°/s; 15°/s] contralateral reflectivity and [30%; 70%] ipsilateral deficit, indicating partial compensation; (T) Total Contralateral Inhibition Profile, with ≤2°/s reflectivity and ≥70% ipsilateral deficit, reflecting substantial contralateral input inhibition and maximal subcortical compensation; (I) Inhibition without Deficit Profile, with ≤15°/s reflectivity and ≤30% ipsilateral deficit, suggesting reduced contralateral reactivity despite a minor deficit.

The regression of the variation of the total energy (E) measurement in static (St) condition with visually controlled condition (VC) for ML balance is significant (*P* < 0.01) and accounts for 43% of the variance. The model shows a causality of dEPN31TS and the SoC component I at the 5% threshold. All results are presented in Table 11.

**Table 11:** OLS *Δ*EStVCML.

Variables	Coefficient Beta	Confidence Intervals	*P*-value
Constant	222	[−1.8; 445]	0.058*
dSM	−1.4	[−4.0; 1.2]	0.3
dSE	−4.5	[−11; 2.3]	0.193
dEPN31TS	−8.4	[−17; 0.07]	0.052*
dSF36BE	18	[7.0; 30]	<0.01***
EdC			<0.01***
N			
I	171	[67; 276]	<0.01***
P	−17	[−107; 72]	0.708
T	31	[−81; 142]	0.59
Model global statistics	R^2^: 0.427	Fisher statistics: 4.800	Fisher test (*p*-value): < 0.010***

* Trend towards statistical significance (*P*-value > 0.05).

** Moderate statistical significance (0.01 < *P*-value ≤ 0.05).

*** Strong statistical significance (*P*-value ≤ 0.01).

**Reading key:** *Δ*EStVCML = *β*(0)+*β*1 × dSM+*β*2 × dSE+*β*3 × dEPN31TS+*β*4 × dSF36BE+*β*5 × EdCI+*β*6 × EdCN+*β*7 × EdCP+*β*8 × EdCT. dSM = Memory dimension of the VestiQ-VS questionnaire, dSE = Emotional dimension of the VestiQ-VS questionnaire, dEPN31TS = Surprise dimension of the EPN 31 questionnaire, dSF36BE = Emotional well-being dimension of the SF36 questionnaire, State of Compensation (SoC) classifications: (N) Non-Inhibited Profile with ≥15°/s contralateral reflectivity and ≤30% ipsilateral deficit, showing no subcortical arc modulation; (P) Partial Contralateral Inhibition Profile, with [2°/s; 15°/s] contralateral reflectivity and [30%; 70%] ipsilateral deficit, indicating partial compensation; (T) Total Contralateral Inhibition Profile, with ≤2°/s reflectivity and ≥70% ipsilateral deficit, reflecting substantial contralateral input inhibition and maximal subcortical compensation; (I) Inhibition without Deficit Profile, with ≤15°/s reflectivity and ≤30% ipsilateral deficit, suggesting reduced contralateral reactivity despite a minor deficit.

The regression of the variation of the total energy (E) measurement in dynamic (D) condition with eyes closed (EC) for ML balance is significant (*P* < 0.01) and accounts for 32% of the variance. The model shows a causality of dimensions dSM, dEPN31J, and dBIG5E at the 5% threshold. All results are presented in Table 12.

**Table 12:** OLS *Δ*EDECML.

Variables	Coefficient beta	Confidence intervals	*P*-value
Constant	−108	[−358; 142]	0.401
dSM	−7.2	[−0.11; 14]	0.047**
dEPN31J	−9.3	[−17; −2.2]	0.011**
dSF36FP	−1.3	[−2.7; 0.23]	0.098*
dBIG5E	13	[5.2; 22]	<0.01***
Model global statistics	R^2^: 0.324	Fisher statistics: 5.270	Fisher test (*p*-value): < 0.010***

* Trend towards statistical significance (*P*-value > 0.05).

** Moderate statistical significance (0.01 < *P*-value ≤ 0.05).

*** Strong statistical significance (*P*-value ≤ 0.01).

**Reading key:** *Δ*EDECML = *β*(0)+*β*1 × dSM+*β*2 × dBIG5E+*β*3 × dEPN31J+*β*4 × dSF36FP. dSM = Memory dimension of the VestiQ-VS questionnaire, dBIG5E = Extraversion, Energy, Enthusiasm dimension of the BFI questionnaire, dEPN31J = Joy dimension of the EPN31 questionnaire, dSF36FP = Physical Functioning dimension of the SF36 questionnaire. State of Compensation (SoC) classifications: (N) Non-Inhibited Profile with ≥15°/s contralateral reflectivity and ≤30% ipsilateral deficit, showing no subcortical arc modulation; (P) Partial Contralateral Inhibition Profile, with [2°/s; 15°/s] contralateral reflectivity and [30%; 70%] ipsilateral deficit, indicating partial compensation; (T) Total Contralateral Inhibition Profile, with ≤2°/s reflectivity and ≥70% ipsilateral deficit, reflecting substantial contralateral input inhibition and maximal subcortical compensation; (I) Inhibition without Deficit Profile, with ≤15°/s reflectivity and ≤30% ipsilateral deficit, suggesting reduced contralateral reactivity despite a minor deficit.

The regression of the variation of the total energy (E) measurement in dynamic (D) condition with visually controlled condition (VC) for ML balance is significant (*P* < 0.01) and accounts for 27% of the variance. The model shows a causality of dimensions dSC, dSE, and dBIG5A at the 5% threshold. All results are presented in Table 13.

**Table 13:** OLS *Δ*EDVCML.

Variables	Coefficient beta	Confidence intervals	*P*-value
Constant	−108	[−364; 41]	0.401
dSF36FP	0.18	[0.46; 0.81]	0.587
dEPN31	−2.3	[−5.5; 0.89]	0.159
dSC	−4	[−7.9; 0.1]	0.044**
dBIG5A	4.9	[−2.9; 9.6]	0.037**
dSE	−5.4	[−9.9; 0.9]	0.019**
Model global statistics	R^2^: 0.272	Fisher statistics: 4.114	Fisher test (*p*-value): < 0.010***

* Trend towards statistical significance (*P*-value > 0.05).

** Moderate statistical significance (0.01 < *P*-value ≤ 0.05).

*** Strong statistical significance (*P*-value ≤ 0.01).

**Reading key:** *Δ*EDVCML=*β*(0)+*β*1 × dSC+*β*2 × dSE+*β*3 × dEPN31J+*β*4 × dBIG5A+*β*5 × dSF36RF. dSC = Cognition dimension of the VestiQ-VS questionnaire, dSE = Emotional dimension of the VestiQ-VS questionnaire, dEPN31J = Joy dimension of the EPN31 questionnaire, dBIG5A = Agreeableness, Altruism, Affection dimension of the BFI questionnaire, dSF36FP = Physical Functioning dimension of the SF36 questionnaire.State of Compensation (SoC) classifications: (N) Non-Inhibited Profile with ≥15°/s contralateral reflectivity and ≤30% ipsilateral deficit, showing no subcortical arc modulation; (P) Partial Contralateral Inhibition Profile, with [2°/s; 15°/s] contralateral reflectivity and [30%; 70%] ipsilateral deficit, indicating partial compensation; (T) Total Contralateral Inhibition Profile, with ≤2°/s reflectivity and ≥70% ipsilateral deficit, reflecting substantial contralateral input inhibition and maximal subcortical compensation; (I) Inhibition without Deficit Profile, with ≤15°/s reflectivity and ≤30% ipsilateral deficit, suggesting reduced contralateral reactivity despite a minor deficit.

#### 3.4.3 Evolution of the angulation of the bisector relative to verticality in the SVV examination

The regression of the bisector angle (Ab) of the dynamic subjective visual vertical (SVVd) is significant (*P* < 0.01) and accounts for 38% of the variance. The model shows causality of the instrumental indicators VORprep and CORg at the 5% threshold. All results are displayed in Table 14.

**Table 14:** OLS regression analysis for dynamic SVV bisector angle change (*Δ*AbSVVd).

Variables	Coefficient beta	Confidence intervals	*P*-value
Constant	−2	[−4.6; 0.59]	0.137
VVORprep	−1.7	[−4.3; 0.87]	0.195
VORprep	1.9	[0.95; 2.9]	<0.01***
IFOg	−8.1	[−24; 7.5]	0.309
CORg	11	[4.6; 17]	<0.01***
Presence of abnormal absolute preponderance (PA)	No		0.275
	Yes	−0.99	[−2.8; 0.79]
Model global statistics	R^2^: 0.375	Fisher statistics: 6.363	Fisher test (*p*-value): < 0.01***

* Trend towards statistical significance (*P*-value > 0.05).

** Moderate statistical significance (0.01 < *P*-value ≤ 0.05).

*** Strong statistical significance (*P*-value ≤ 0.01).

**Reading Key:** *Δ*AbVVSd=*β*(0)+*β*1 × VVORprep+*β*2 × VORprep+*β*3 × IFOg+*β*4 × CORg+*β*5 × PAA+*β*6 × PAN. VVORprep = Preponderance observed during the sensitized burst test for the visuo-vestibulo-ocular reflex study.

VORprep = Preponderance observed during the sensitized burst test for the vestibulo-ocular reflex study.

IFOg = Gain obtained during the sensitized burst test for the study of the ocular fixation index.

CORg = Gain obtained during the sensitized burst test for the study of the cervico-ocular reflex index.

PAA = Abnormal absolute preponderance (≥2°/s) in the bithermal test.

PAN = Normal absolute preponderance (≤2°/s) in the bithermal test.

## 4 Discussion

### 4.1 Cohort presentation

The findings of this study highlight several important points regarding the population recruted, clinical follow-up their clinical significance. Patients were included over two consecutive years, with a slight predominance in 2021 (59.7%) compared to 2022 (40.3%). The average follow-up duration was 13 months, with an average of 87 rehabilitation sessions. The patients' professional distribution showed diversity, with a majority being retirees (46.8%; Table 3).

Analysis of initial and final diagnoses of patients revealed significant changes during the rehabilitative care. For instance, 8.1% of the cohort was diagnosed with central disorders after the beginning of rehabilitation, while the initial diagnosis of recurrent BPPV decreased from 33.9% to 9.7% by the end of rehabilitation. Moreover, 24.2% of unspecific vestibular vertigos were diagnosis by the end of care. These results underline the importance of clinical reevaluation to improve diagnosis according to clinical changes during rehabilitation program.

Regarding visual symptoms, the study found significant changes between the first vertigo crisis and the first integrative vestibular rehabilitation therapy (iVRT) consultation. For example, visual fatigue increased from 4.8% to 38.7% of the cohort, and movement-induced blurred vision increased from 11.3% to 59.7% of the cohort during the first iVRT consultation (Table 4). These results suggest an evolution of visual symptoms in patients with chronic vertigo (CVP), underlying compensation mechanisms. which could have significant implications for iVRT management in terms of intervention.

Finally, regarding associated syndromes such as chronic neck pain (CN) and temporomandibular disorders (TMD), 8% of the cohort suffered from CN before the first crisis compared to 13% at inclusion, and 6.5% from TMD compared to 14.5% at inclusion.

### 4.2 The action of iTRV: questionnaire analysis

In our study, significant improvements were observed post iVRT in various questionnaires assessing the impact of vertigo on quality of life. The DHI (Supplementary Table S9) revealed a significant decrease in emotional scores from 45.31 to 28.57 and functional scores from 50.00 to 29.17 (*p* < 0.05), indicating an improvement in the perception of handicap related to vertigo. The SF36 (Supplementary Table S9) showed improvements of physical level (from 62.41 to 76.34) and physical health limitations (from 68.10 to 52.68), suggesting an enhancement in physical quality of life (*p* < 0.05). Particularly notable was the improvement of mental health, with an increase from 50.55 to 58.07 of the emotional well-being dimension after iVRT (*p* < 0.05). The EPN-31 results (Supplementary Table S9) indicate an improvement in joy (from 19.69 to 23.80) and a reduction in shame (from 39.87 to 18.88), demonstrating a positive impact on emotions (*p* < 0.05). Similarly, the Big Five Inventory (BFI; Supplementary Table S9) revealed an increase in extraversion after iVRT (from 3.16 to 2.76, *p* < 0.05). The VestiQ-VS (Supplementary Table S9) showed a significant improvement of psychological state (from 47.31 to 27.64) and emotional state (from 39.87 to 24.55), confirming the efficacy of iVRT on the psychological and emotional state (*p* < 0.05).

However, certain dimensions like pain in the SF36 and memory in the VestiQ-VS did not show significant change, suggesting that iVRT does not directly affect these aspects (Supplementary Table S9). Despite overall improvements, specific emotional and physical limitations persist (shown by the SF36 dimensions), possibly influenced by external factors not evaluated in this study.

### 4.3 Study of instrumental tracking indicators

This section discusses the relevance of tracking indicators in chronic vestibular patients (CVP) beyond the notions of normality often attributed to instrumental examinations, which are necessary in clinical conditions dealing with acute cases as well as pre- and post-surgical monitoring. However, it seems, based on our results, that CVP impacts vestibular function differently in the presence of a permanent and/or recurrent error signal. The focus of our approach is on the notion of vestibular error signal (VES), which is of paramount importance in addressing the patient in rehabilitation. We know that a supraliminal VES not only induces consequences on the behavioral performance of the VOR but also adaptive consequences through the central compensation capacities at subcortical and cortical levels (3, 45–47) and strategy of the sensori-perceptual-motor (SPM) system (23, 48). What we are beginning to understand is that a weak or subliminal VES also induces behavioral responses (9) and causes errors in spatial orientation during mental imagery tasks (46). The integration of the VES and its study appear to define subcategories of adaptation and SPM response, some of which have been recorded during our work. These are developed in the following subsections.

#### 4.3.1 Posturography indicator analysis

Our study demonstrates significant improvements (*p* < 0.05) in vestibular function and mediolateral (ML) composite scores after iVRT, underscoring the effectiveness of iVRT on these aspects, even in older subjects. These results support the established links between vestibular function and ML balance found in the literature (49, 50). Although variations in other posturography scores were noted, they were not statistically significant, highlighting the sensitivity and potential for false positives in the algorithmic methods used for analysis. This raises questions about the specificity and interpretation of posturography measurements in CVP and suggests integrating functional tests of the vestibulo-ocular reflex for a more sensitive analysis, as recommended by Di Fabio (27). This is what we have proposed to the reader in the following sections.

#### 4.3.2 Analysis of indicators from kinetic VNG tests

##### 4.3.2.1 Analysis of VOR2 gain (VOR2g) and COR gain (CORg)

Regarding the indicators from kinetic VNG, the analysis showed mixed results. Not all variables studied demonstrated significant differences between A1 and A2 in terms of normalization changes, indicating that rehabilitation does not seem to have a direct impact on reflectivity (preponderance). However, the analysis of value variations according to improvement or deterioration towards normalization was significant as shown in the kinetic evaluation of the vestibulo-ocular reflex gain sensitized in a dual mental task (VOR2g) and the kinetic evaluation of the cervico-ocular reflex gain (CORg). This might support the evolution of compensation in these patients, not related to a restoration of the peripheral function of the vestibular system but indeed related to a more complex modulation of the sensori-perceptual-motor (SPM) system.

##### 4.3.2.2 Comparative analysis of VOR (VORg) and VOR2 (VOR2g) gain trends

The interpretation of VOR2 gain depends on the value of VOR gain. Generally, an improvement in VOR2 gain could express central disinhibition in CVPs, but when it deteriorates, the interpretation becomes dependent on the clinical context. A VOR2 gain approaching the VOR gain seems to express the absence of inhibition (condition 2; Table 15).

**Table 15:** Interpretation of gains in kinetic videonystagmography (VNGc) burst test.

Condition	Increase in VOR2g (n)	Decrease in VOR2g (n)	Gain Ratio between VOR and VOR2	Interpretation
Condition 1	14	12	VORg < VOR2g	Inhibition
Condition 2	2	5	VORg≈VOR2g	No Inhibition
Condition 3	8	2	VORg > VOR2g	Context Dependent

This table provides insights into the kinetic videonystagmography (VNGc) burst test's outcomes, categorizing patients based on the changes in their vestibulo-ocular reflex gain (VOR2g). Condition 1 indicates (in)voluntary inhibition where the gain of the reflex in a dual task (VOR2g) is lower than the standard reflex gain (VORg), suggesting a dampening effect. Condition 2 reflects a scenario with no significant inhibition, where the gains are approximately equal, indicating normal function. Condition 3's interpretation depends on the clinical context, suggesting potential overcompensation or a cognitive threshold effect where VORg surpasses VOR2g, possibly indicating an adaptive or maladaptive response to vestibular stimuli.

Among the 26 patients identified in condition 1 (Table 15), 14 improved. Regarding condition 3, it is revealing for us, in chronic patients, of a plateau effect already questioned in the literature (51–54). The decrease of a gain in a dual task might indicate the presence of a cognitive task difficulty threshold beyond which the patient becomes less efficient at the vestibular level. This observation aligns with those presented by Xavier et al. (10) in patients with vestibular schwannoma. The evolution of the fatigue component of the VestiV-QS is very explicit: among the 14 patients who present an increase in VOR2 gain, fatigue improves significantly compared to the 12 patients who saw their VOR2 gain decrease. Future research should delve deeper into these observations and further explore the underlying mechanisms of these evolutions. Nonetheless, we suggest monitoring the fatigue indicator before, during, and 48 h after iVRT. However, unlike concussions where specific scales like the Post-Concussion Symptom Scale (PCSS) are commonly used to assess symptoms and fatigue, there are no standardized equivalent tools for vestibular disorders (55). Measuring neurological fatigue can be complex, as it depends on many factors specific to each patient and their neurological condition. Health professionals may use a combination of tools and methods to assess neurological fatigue, including: (i) questioning symptoms: doctors and therapists can perform subjective clinical assessments to evaluate the patient's neurological fatigue based on their observations and the patient's reports; (ii) using measurement scales: some general fatigue measurement scales such as the Chalder Fatigue Scale (56) can be adapted for patients with chronic vestibular disorders to assess their fatigue; (iii) tracking symptoms and performance: regular monitoring of the patient's symptoms and their performance on specific tasks in iVRT can also help assess neurological fatigue.

##### 4.3.2.3 Analysis of reflectivity from the thermal VNG test

Notable variations in reflectivity were observed in some subgroups, with significant improvements and deteriorations, indicating individual changes in reflectivity independently of the overall association with rehabilitation. Cases of reflectivity lateralization reversal after rehabilitation were noted, requiring specific analysis for their clinical implications. These findings reveal the complexity of the impact of rehabilitation on reflectivity and emphasize the importance of future studies to explore these variations in detail and identify possible beneficial interventions for vestibular patients.

##### 4.3.2.4 Analysis of composite indicators

The analysis of the hyperactive vestibular error signal (SH) showed a resolution of this signal within the cohort studied in A2. Due to the retrospective nature of our study, we were not able to identify the origin of this signal. However, given that the studied population consists of chronic patients (i.e., presenting persistent symptoms a year after the crisis, at a minimum to be included in the study), we can suggest a multifactorial origin resolved through our integrative program.

The analysis of the evolution of vestibular compensation (SoC) through the state of reflectivity of the healthy ear when available is a relevant follow-up indicator already proven in the literature (57, 58). These articles show that VRT has a significant impact on acute vestibular patients and even on certain profiles of instrumental areflexia that can improve after treatment. However, in the context of CPV, SoC seems to evolve differently. Indeed, SoC showed discrete changes between the beginning (A1) and the end of iVRT (A2). The results revealed that 46.6% of the cohort had an absence of compensation in the caloric test (N) between A1 and A2, but, examining the details of the fluctuations, 3 among the 28 patients in this group migrated to a SoC category that may indicate the presence of a subliminal error signal (I) and 1 to a moderate compensation due to a deficient VES (P), while 2 moved from category P to N and 2 from category I to N. Additionally, 6.7% of the cohort showed strong compensation that may result from a strong deficient VES (T profile) in A2, compared to 1.7% in A1. In total, 18 patients (30%) observed fluctuation in the bithermal test, including 3 with progressive deterioration of the instrumental vestibular signal. 24 patients did not fluctuate, remaining in a SoC N category and forming a homogeneous group until A2. This last observation may indicate that over a period of iVRT management, the state of vestibular compensation of patients is not acquired for 70% of the cohort. Moreover, apart from the 3 central diagnoses corresponding to the 3 patients who shifted from an I state to a T state, other fluctuations seem impacted by iVRT. Given the restricted numbers of the subgroups, further studies are necessary to determine if the rehabilitation did not have a deleterious effect, especially for the 4 patients who exited a SoC N category: a single case study will be proposed later.

##### 4.3.2.5 Analysis of subjective visual vertical (SVV**)**

The notion of precision and accuracy is an essential prerequisite in the study of somatosensory signals. The concept of precision within the framework of SVV is widely addressed in the literature (25, 59). Our innovation was to introduce the notion of precision and accuracy into the spatial modeling of our measurements. In our study, the analysis of subgroups (improvement, deterioration) reveals distinct trends. It is important to note that these results show a significant variation in the value of the geometric angle (obtained by averaging the measurements taken from the right and left tilt starting points) and not in the value of the bisector angle relative to the vertical axis. This could correspond to a modulation of precision (observed through the variation in the geometric angle) rather than a variation in accuracy between A1 and A2 (Table 4). This reinforces the idea that rehabilitation impacts the sensorimotor-perceptual (SPM) reference frame, allowing the central nervous system to integrate other information (such as somesthetic information) by modulating the weight of different sensory signals and thus optimizing precision, modeled by the reduction of the geometric angle in A2. This new SVV analysis opens perspectives for observing the establishment of multisensory integrative compensation achieved after iVRT.

Our study examined the impact of several factors on SVV in subjects undergoing iVRT, analyzing the influence of CORg, SoC profiles, and the presence of an SH (Table 5). For the composite variable SH, the coefficients of variation (CV) and Gini values (Cg) are lower in the group without SH for the measurement of the static and dynamic SVV geometric angles. This suggests a certain homogeneity in the variations of these angles in this group. The geometric angle improvements observed in the group without SH between A1 and A2 seem to reach a higher proportion of patients compared to the deterioration group, while the group with SH, showing a higher proportion of deterioration, is mainly affected in the dynamic geometric angle measurement. In conclusion, the increase in disparities in geometric angle measurements and the degradation observed for 73% of the SH group in dynamic conditions suggest increased difficulty in SPM performance for these patients, especially when subjected to induced conflict during 20°/s optokinetic stimulation. We observe that the strategy used to resolve the imprecision is to switch to optimal accuracy performance, identified in our study by increased homogeneity of the SVV bisector angle measurement in dynamic conditions in the group where SH is present. Our hypothesis is that the central nervous system, in the context of SPM adjustment in response to a chronic hyperactive vestibular error signal (VES), would be more “rigid” and less inclined to modulate the confidence interval of the extreme SVV measurements. In other words, the strategy of optimizing precision is less effective in this context. The optimization of accuracy recruitment strategies seems more complex to modulate. Our hypothesis is that there is a strong link between accuracy and the internal model. The strategy of accuracy modulation seems useful in the presence of imprecision. However, this strategy has limits because when the internal model is biased, the strategy of enhancing precision is ineffective, as demonstrated in the case of “pushers” (60). We hypothesize that accuracy is moderately biased by the internal model in CPV patients subjected to a chronic hyperactive VES. This is why in CPV, uTRV proposes scenarios with the notion of useful error: the patient is subjected to a progression of exercises in which they experience error progressively until reaching a maximum threshold beyond which the patient will experience a return of symptoms. This is a well-known rehabilitative profile in the management of concussions (61, 62).

Patients whose SoC evolved during iVRT show more homogeneous geometric angle measurements in dynamic SVV conditions, suggesting the use of this strategy during variations in reflectivity and deficit, thus linking the quality of peripheral signal integration to that of SPM integration. For CORg, there is a clear link between the variation in COR gain and the homogeneity of the results obtained for SVV (Table 5). The values of geometric angles in static and dynamic conditions are more homogeneous in patients who did not experience a variation in COR gain during iVRT, strongly suggesting the involvement of vestibulo-collic pathways among the possible SPM compensation strategies (60–62). It appears that the recruitment of cervical proprioceptive inputs impacts the accuracy of SVV measurements in CPV patients.

##### 4.3.2.6 Analysis of optometric indicator results

The analysis of optometric indicators yielded very interesting results. The significant improvement in near visual acuity (NVA) post-iVRT was unexpected as it has not been presented in the literature and, given the age of our cohort, was expected to show a trend towards deterioration. This highlights, for us, the potential effect of our intervention not only on balance and vestibular function but also on more global aspects such as psychic, neurovisual, and locomotor aspects. Indeed, our care has evolved with sequences (Table 1) focused on a integrative approach including osteo-articular aspects for the approach of temporomandibular and cranio-cervical dysfunctions, neurovisual for fusion disorders, and psycho-behavioral for mood disorders. This significant improvement from a statistical standpoint (*P* < 0.01) could reflect the complex interdependence between SPM integration and the notion of chronicity.

The results of prismatic analyses, although not significant, suggest that iVRT does not negatively interfere with binocular vision, a fundamental aspect for near visual acuity.

The results obtained in the Mawas board (PmW) examination show that significant variations in fusion were measured between 15 and 20 cm from nasion. The variation at 25 cm could also be considered (*p* = 0.10) and re-evaluated in another study. Here again, iVRT seems to significantly influence the neurosensory aspect of near vision.

The study of the near point of accommodation (NPA) shows a significant evolution between A1 and A2 (*p* < 0.05) with two groups either improving or diminishing in performance.

The study of distance stereograms shows a significant change in the presentation of the star, cat, and car at one meter, as well as the presentation of the circle and star at five meters. These results suggest that iVRT may impact patients' spatial perception when it integrates the use of stereograms specific to our research work.

Finally, the examination with the Worth lamp confirms these results, for which an improvement in stereoscopic vision is observed for 60% of the cohort (*p* = 0.029).

Visual fusion, dependent on the horopter and Panum's area, is an essential mechanism for three-dimensional perception. In the context of vestibular asthenopia, the associated spatial disorientation can lead to disturbances in visual fusion, exacerbating visual symptoms. Integrating the neurovisual sphere in concepts of rebalancing, facilitation, and sensori-perceptual-motor reprogramming in our treatment sequences is one of the strengths of our approach. These observations corroborate the results obtained by Xavier et al. (9) in a previous study showing that subliminal VES impacts the visuo-oculomotor component. It is highly probable that the management of chronic VES benefits from similar resolution mechanisms, impacting subtle aspects of vision such as fusion and stereoscopy.

### 4.4 Study of predictive markers

In this section, we discuss the predictive markers we have identified in our study. It seems useful to search for these markers to best impact the effects of physical therapy.

#### 4.4.1 Study of predictive markers of medio-lateral stability

**Figure 10:**
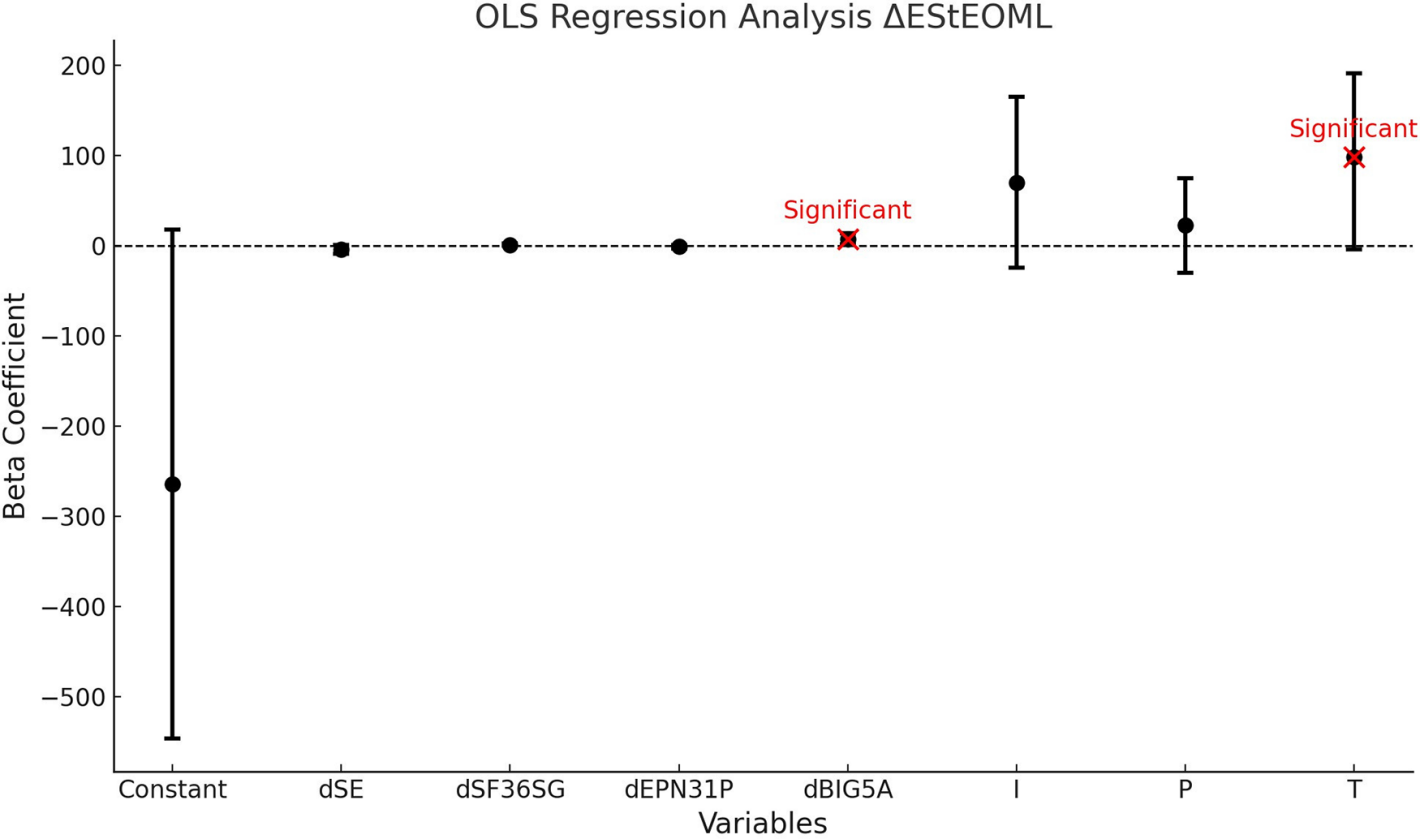
Ordinary least squares (OLS) regression analysis EStEOML. This graph shows the beta coefficients of the variables used in the OLS regression analysis for the dynamic change in the EStEOML. The beta coefficients indicate the strength and direction of the association between each variable and the dynamic EStEOML. Figure Components: • Black Dots: Each black dot represents a beta coefficient for a given variable. • Error Bars: The horizontal bars around the dots indicate the 95% confidence intervals for each beta coefficient. They show the range within which the true beta coefficient is likely to lie with a 95% probability. • Red Dots: Red dots indicate variables whose beta coefficients are statistically significant (*p* < 0.05). Significant variables are annotated with the text “Significant”. • Horizontal Dashed Line at Zero: The dashed line indicates the zero value of the beta coefficient. A beta coefficient of zero means there is no association between the variable and the dynamic EStEOML How to Read the Figure: • Identify the Variables: The variables are listed on the x-axis. They include “Constant”, “dSE”, “dSF36SG”, “dEPN31P”, “dBIG5A”, “I”, “P”, and “T”. • Understand the Coefficients:The position of the black dots on the y-axis represents the beta coefficients for each variable. A positive coefficient indicates a positive association with EStEOML, while a negative coefficient indicates a negative association. Evaluate Significance: • Look at the red dots to identify significant variables. These variables have a statistically significant association with EStEOML. • Error bars that do not cross the horizontal dashed line at zero also indicate significance. Variable Definitions: dSE: Emotional dimension of the VestiQ-VS questionnaire, dSF36SG: General health dimension of the SF36 questionnaire, dEPN31P: Fear dimension of the EPN31 questionnaire, dBIG5A: Agreeableness, Altruism, Affection dimension. I: Inhibition without Deficit Profile, P: Partial Contralateral Inhibition Profile, T: Total Contralateral Inhibition Profile.

**Figure 11:**
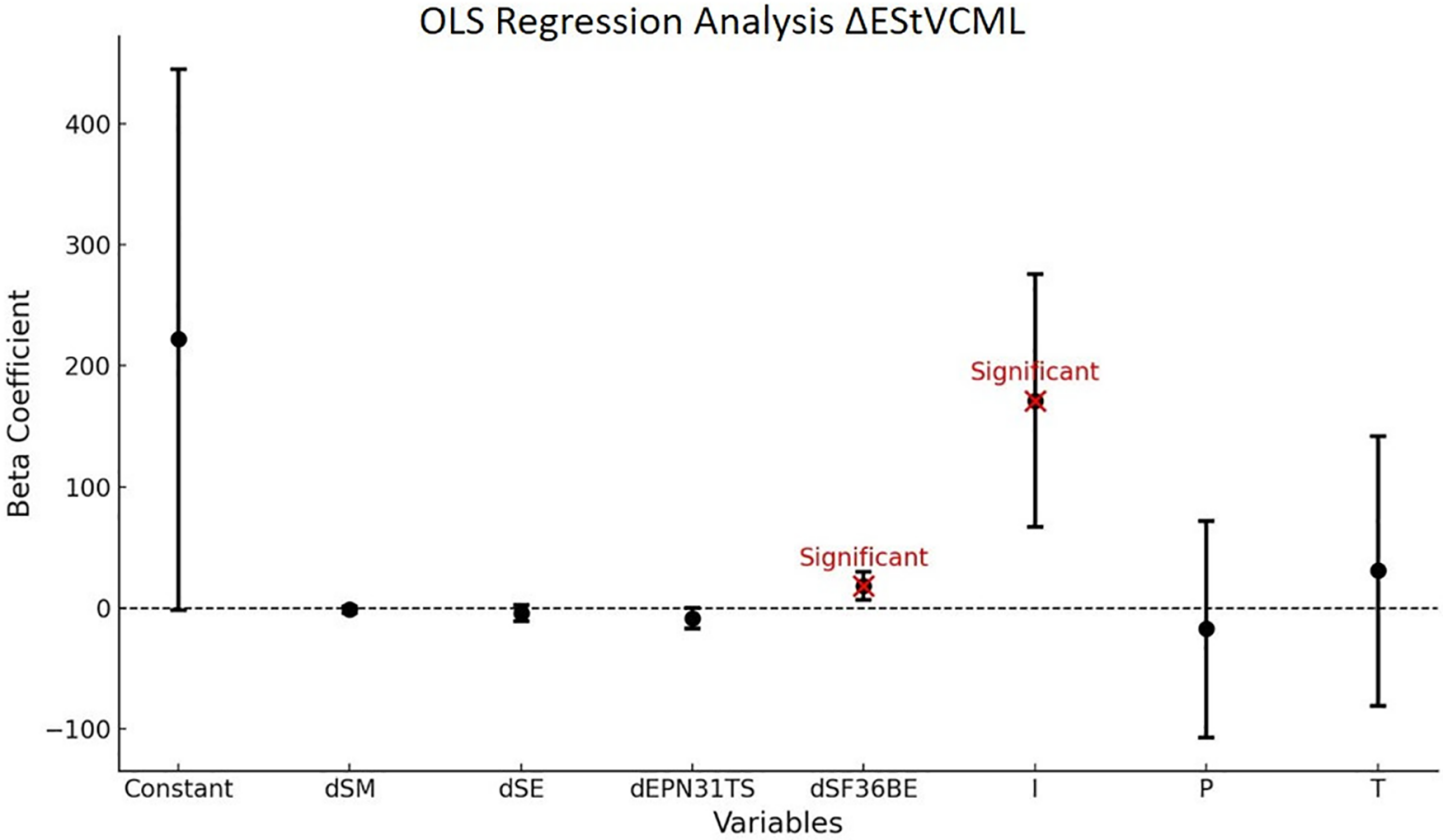
Ordinary least squares (OLS) regression analysis EStVCML. This graph shows the beta coefficients of the variables used in the OLS regression analysis for the dynamic change in EStVCML. The beta coefficients indicate the strength and direction of the association between each variable and the dynamic EStVCML. Figure Components: • Black Dots: Each black dot represents a beta coefficient for a given variable. • Error Bars: The horizontal bars around the dots indicate the 95% confidence intervals for each beta coefficient, showing the range within which the true beta coefficient is likely to lie with 95% probability. • Red Dots: Red dots indicate variables whose beta coefficients are statistically significant (*p* < 0.05). Significant variables are annotated with the text “Significant”. • Horizontal Dashed Line at Zero: The dashed line indicates the zero value of the beta coefficient. A beta coefficient of zero means there is no association between the variable and EStEOML. How to Read the Figure: • Identify the Variables: The variables are listed on the x-axis. These include “Constant,” “dSM,” “dSE,” “dEPN31TS,” “dSF36BE,” “I,” “P,” and “T”. • Understand the Coefficients: The position of the black dots on the y-axis represents the beta coefficients for each variable. A positive coefficient indicates a positive association with dynamic EStVCML, while a negative coefficient indicates a negative association. Evaluate Significance: • Look at the red dots to identify significant variables. These variables have a statistically significant association with dynamic EStVCML. • Error bars that do not cross the horizontal dashed line at zero also indicate significance. Variable Definitions: dSM: Memory dimension of the VestiQ-VS questionnaire, dSE: Emotional dimension of the VestiQ-VS questionnaire, dEPN31TS: Surprise dimension of the EPN31 questionnaire, dSF36BE: Emotional well-being dimension of the SF36 questionnaire.

**Figure 12:**
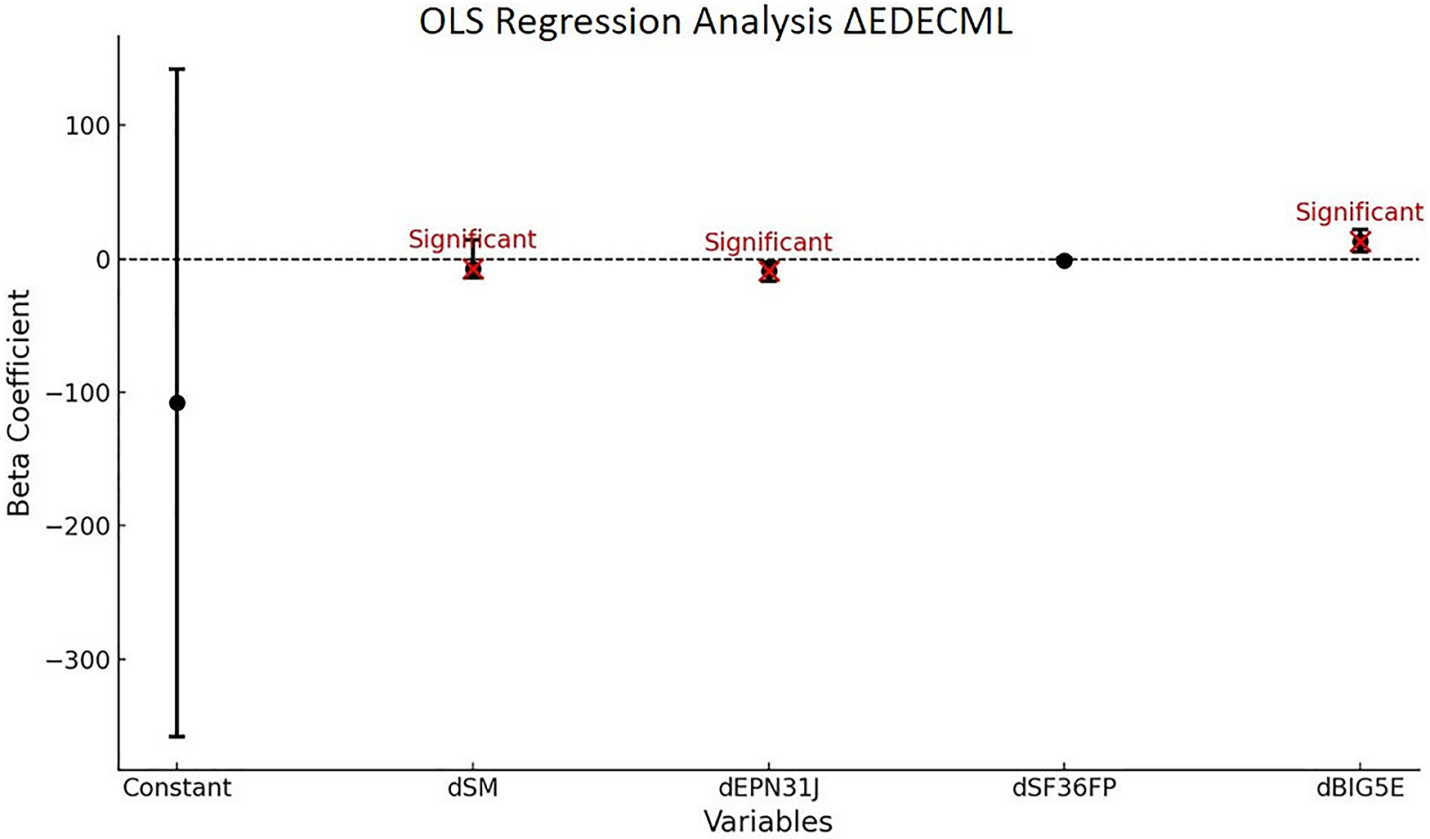
Ordinary least squares (OLS) regression analysis EDECML. This graph shows the beta coefficients of the variables used in the OLS regression analysis for EDECML. The beta coefficients indicate the strength and direction of the association between each variable and EDECML. Figure Components: • Black Dots: Each black dot represents a beta coefficient for a given variable. • Error Bars: The horizontal bars around the dots indicate the 95% confidence intervals for each beta coefficient. They show the range within which the true beta coefficient is likely to lie with a 95% probability. • Red Dots: Red dots indicate variables whose beta coefficients are statistically significant (*p* < 0.05). Significant variables are annotated with the text “Significant”. • Horizontal Dashed Line at Zero: The dashed line indicates the zero value of the beta coefficient. A beta coefficient of zero means there is no association between the variable and EDECML. How to Read the Figure: • Identify the Variables: The variables are listed on the x-axis. They include “Constant”, “dSM”, “dEPN31J”, “dSF36FP”, and “dBIG5E”. • Understand the Coefficients: The position of the black dots on the y-axis represents the beta coefficients for each variable. A positive coefficient indicates a positive association with EDECML, while a negative coefficient indicates a negative association. Evaluate Significance: • Look at the red dots to identify significant variables. These variables have a statistically significant association with EDECML. • Error bars that do not cross the horizontal dashed line at zero also indicate significance. Variable Definitions: dSM: Memory dimension of the VestiQ-VS questionnaire, dBIG5E: Extraversion, Energy, Enthusiasm dimension of the BFI questionnaire, dEPN31J: Joy dimension of the EPN31 questionnaire, dSF36FP: Physical Functioning dimension of the SF36 questionnaire.

**Figure 13:**
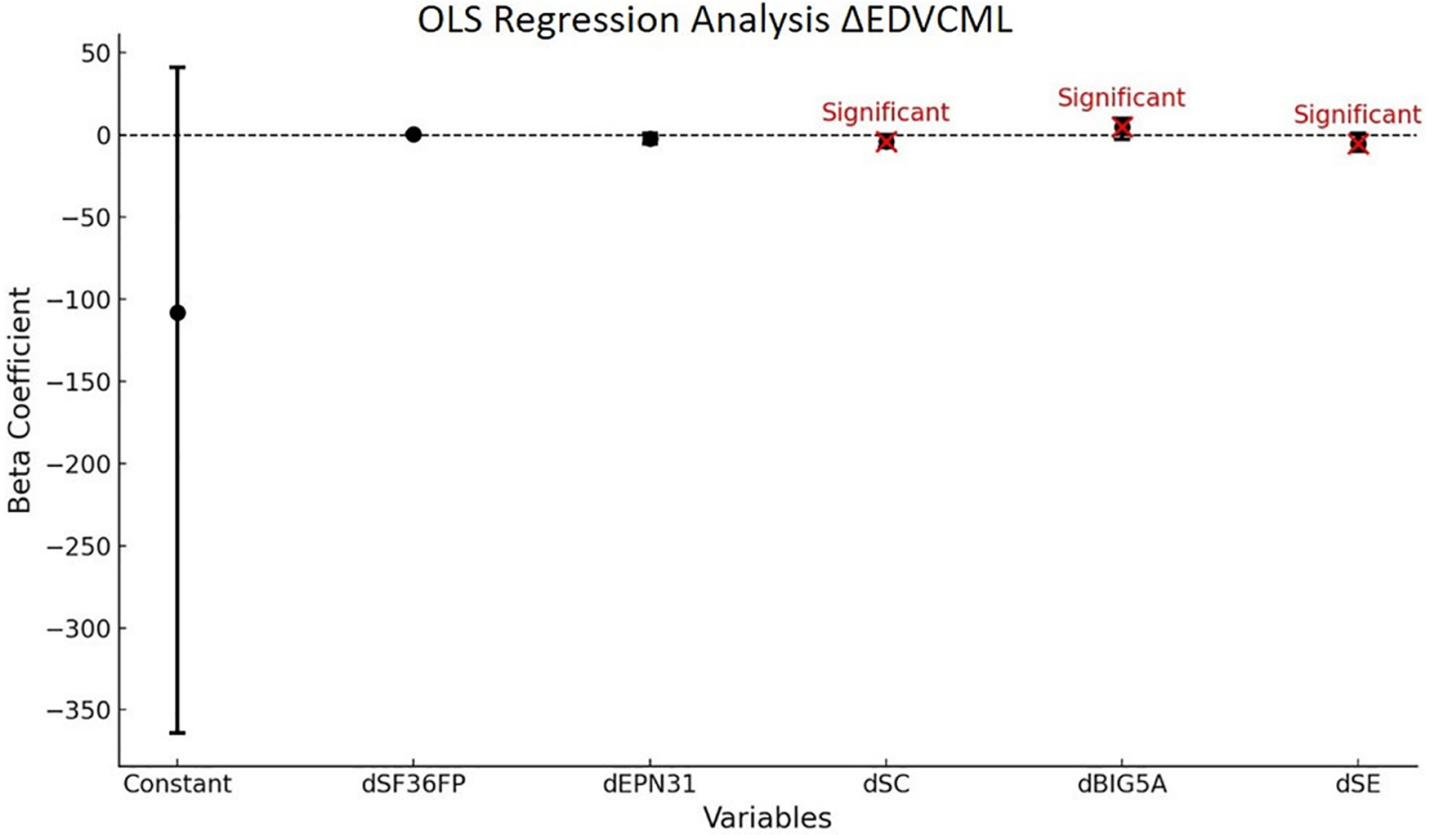
Ordinary least squares (OLS) regression analysis EDVCML. This graph shows the beta coefficients of the variables used in the OLS regression analysis for EDVCML. The beta coefficients indicate the strength and direction of the association between each variable and the outcome measure (EDVCML). Figure Components: • Black Dots: Each black dot represents a beta coefficient for a given variable. • Error Bars: The horizontal bars around the dots indicate the 95% confidence intervals for each beta coefficient. They show the range within which the true beta coefficient is likely to lie with a 95% probability. • Red Dots: Red dots indicate variables whose beta coefficients are statistically significant (*p* < 0.05). Significant variables are annotated with the text “Significant”. • Horizontal Dashed Line at Zero: The dashed line indicates the zero value of the beta coefficient. A beta coefficient of zero means there is no association between the variable and the outcome measure (EDVCML). How to Read the Figure: • Identify the Variables: The variables are listed on the x-axis. They include “Constant”, “dSF36FP”, “dEPN31”, “dSC”, “dBIG5A”, and “dSE”. • Understand the Coefficients: The position of the black dots on the y-axis represents the beta coefficients for each variable. A positive coefficient indicates a positive association with EDVCML, while a negative coefficient indicates a negative association. Evaluate Significance: • Look at the red dots to identify significant variables. These variables have a statistically significant association with EDVCML. • Error bars that do not cross the horizontal dashed line at zero also indicate significance. Variable Definitions: dSC: Cognition dimension from the VestiQ-VS questionnaire, dSE: Emotional dimension from the VestiQ-VS questionnaire, dEPN31J: Joy dimension from the EPN31 questionnaire, dBIG5A: Agreeableness, Altruism, Affection dimension from the BFI questionnaire, dSF36FP: Physical functioning dimension from the SF36 questionnaire.

Vestibular signals play a crucial role in maintaining upright posture, especially under unstable postural conditions where other sources of sensory information are diminished or absent. They are particularly involved in detecting and correcting rapid and significant postural movements (63). Vestibulo-spinal reflexes are modulated based on postural conditions and play a role in posture adjustment to maintain stability, especially in the ML plane (63). Studying the underlying mechanisms of ML balance is significantly important for our understanding of postural control and human mobility, especially in vulnerable populations such as patients with chronic vestibular instability and symptoms (64). Complex processes are involved in maintaining ML balance during essential tasks such as transitioning from sitting to standing or in instability situations with rapid fluctuations in the ML plane (65). Previous studies have suggested that ML balance may be more sensitive to sensory disturbances and age-related alterations than anteroposterior (AP) balance. It is known that anxiety states affect postural performance (66). Similar to studies in the field, our study was able to determine a predictive link between cognitive-emotional and psycho-behavioral health and balancing performance in the mediolateral plane. We were able to specify the impact of different factors studied through the dimensions of the questionnaires used in our study. The analysis of total energy variation in 4 ML conditions revealed several key findings under:

• Static, Eyes Open (Table 10, Figure 10): A significant relationship (*P* < 0.01) was found, with 31% of the variance explained. The emotional dimension indicates a negative correlation, suggesting that emotional deterioration is related to increased postural imbalance. Conversely, better overall state health is associated with improved stability. Fear and pleasantness dimensions did not show a significant correlation with postural imbalance.
• Static, visually controlled condition (VC; Table 11, Figure 11): A significant correlation (*P* < 0.01) was observed, with 43% of the variance explained. Emotional dysfunctions and imbalance in the experience of surprise are associated with increased instability, while better emotional well-being favors stability. Central compensation levels also influence balance, but memory disorders do not have a significant impact.
• Dynamic, Eyes Closed (EC; Table 12; Figure 12): A significant relationship (*P* < 0.01) with 32% of the variance explained was showed. Unimpaired memory functioning and high levels of extraversion are linked to better stability. However, an imbalance in the experience of joy is associated with increased imbalance, and physical function did not show a significant correlation.
• Dynamic, visually controlled condition (VC; Table 13; Figure 13): A significant relationship (*P* < 0.01) with 27% of the variance explained. Better cognitive abilities and high levels of pleasantness are associated with improved stability. Global emotional dysfunction is linked to increased imbalance, while fluctuations in joy and physical function did not show a significant correlation in this condition.

At this stage, it seems relevant to consider that the difficulty levels in the evaluated imbalance conditions imply different connections with cognitive-emotional (CE) recruitment for each of them. Thus, ranking the Sensory Organization Test tasks by difficulty level should also be discussed by jointly evaluating the CE recruitment capabilities specific to each patient. With the introduction of a cognitive-vestibular system (Lacroix 2021), it is suggested that each patient has a specific threshold beyond which the sensori-perceptual-motor system, and thus the balancing ability in contexts of visual deprivation, balancing performance, sensory conflict, or dual-task situations, fails. This threshold represents the limit beyond which managing balancing conditions becomes too energetically demanding for higher cognitive functions. This phenomenon indicates not only that certain patients with chronic vestibular disorders require an energy-intensive recruitment of higher cognitive functions to maintain balance but also that CE plays a significant role in managing cognitive resource allocation for balancing capabilities in complex situations. Consequently, there is a threshold beyond which managing balancing conditions is no longer ecological, highlighting the need for a personalized therapeutic approach to optimize vestibular compensation and sensory integration, emphasizing the crucial importance of the interaction between CE, the allocation of cognitive resources to the compensation of a chronic VES, and balancing capabilities.

#### 4.4.2 Study of predictive markers of the variation in the inclination of the bisector relative to verticality; of the angle formed by the average of the SVV measurements in dynamic condition (optokinetic at 20°/s)

**Figure 14:**
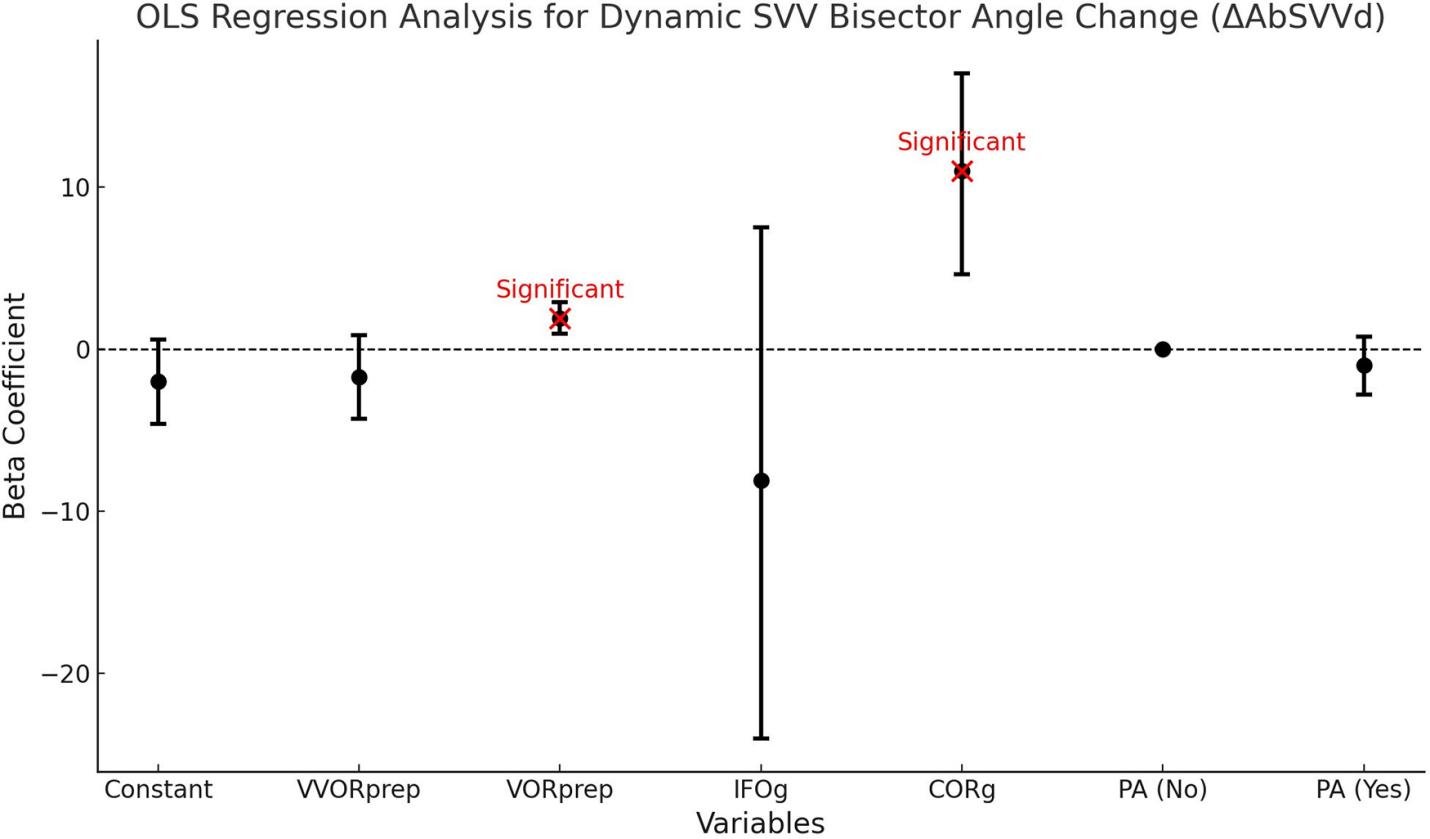
Ordinary least squares (OLS) regression analysis for dynamic SVV bisector angle change (AbSVVd). This graph shows the beta coefficients of the variables used in OLS regression analysis for the dynamic variation of the bisector angle of SVV (AbSVVd). The beta coefficients indicate the strength and direction of the association between each variable and the dynamic change in the SVV bisector angle. Figure Components: • Black Dots: Each black dot represents a beta coefficient for a given variable. • Error Bars: The horizontal bars around the dots indicate the 95% confidence intervals for each beta coefficient. They show the range within which the true beta coefficient is likely to lie with a 95% probability. • Red Dots: Red dots indicate variables whose beta coefficients are statistically significant (*p* < 0.05). Significant variables are annotated with the text “Significant”. • Horizontal Dashed Line at Zero: The dashed line indicates the zero value of the beta coefficient. A beta coefficient of zero means there is no association between the variable and the dynamic bisector angle change of SVV. How to Read the Figure: • Identify the Variables: The variables are listed on the x-axis. They include measures such as “Constant”, “VVORprep”, “VORprep”, “IFOg”, “CORg”, and categories of “Presence of Abnormal Absolute Preponderance (PA)”. • Understand the Coefficients: The position of the black dots on the y-axis represents the beta coefficients for each variable. A positive coefficient indicates a positive association with the dynamic SVV bisector angle change, while a negative coefficient indicates a negative association. • Evaluate Significance: Look at the red dots to identify significant variables. These variables have a statistically significant association with the dynamic SVV bisector angle change. Error bars that do not cross the horizontal dashed line at zero also indicate significance. Variable Definitions: • VVORprep: Preponderance observed during the sensitized burst test for the visuo-vestibulo-ocular reflex study, • VORprep: Preponderance observed during the sensitized burst test for the vestibulo-ocular reflex study, • IFOg: Gain obtained during the sensitized burst test for the study of the ocular fixation index, • CORg: Gain obtained during the sensitized burst test for the study of the cervico-ocular reflex index, • PA (Yes): Abnormal absolute preponderance (≥2°/s) in the bithermal test, • PA (No): Normal absolute preponderance (≤2°/s) in the bithermal test.

Tonal imbalances of the vestibular system, traditionally associated with unilateral peripheral vestibular lesions, have been reevaluated. These studies suggest that beyond otolithic lesions, dysfunctions at different levels of the vestibular system, including spinal, vestibular nucleus, brainstem, interstitial nucleus of Cajal lesions, as well as lesions located above the brainstem, thalamic, and cortical in the insular and temporo-parietal regions, can affect SVV and ML balance. These impairments can lead to complex dysfunctions such as visuospatial hemineglect and pusher syndrome, influencing both cognition and various sensory modalities (60). Furthermore, neural network modeling reveals that SVV inclinations result not only from otolithic imbalances but also from anomalies in the tone of vertical semicircular canals, affecting the central estimation of gravity. This model highlights the importance of the vertical semicircular canal in SVV inclinations, proposing a reevaluation of the causes of vestibular lesions, which would result from combined dysfunction of otoliths and semicircular canal input (60). In our model (Table 14; Figure 14), we also showed a significant relationship (*P* < 0.01) between the variation of the SVV bisector angle in dynamic conditions and the explanatory variables, contributing 38% to the variance. The results indicate that VOR preponderance and COR gain are positively and significantly associated with the variation of SVV inclination. This suggests a strong relationship in chronic vestibular patients between the variation of VOR preponderance and COR gain and that of the SVV angle in conditions of visual disturbance while no significant correlation is observed with VVOR, IFO, and absolute preponderance in the bithermic examination. Thus, the SPM recruitment in some of our chronic patients with instability complaints would be multimodal proprioceptive involving cervical and oculomotor proprioception according to our theory of “short” or short-latency neural networks.

### 4.5 Multisensory modalities

These findings prompt a reevaluation of the underlying mechanisms governing the interaction between the vestibular and visual systems, particularly regarding the processing and integration of sensory information. Vestibular compensation appears to be influenced by two systems: the first involves a non-cognitive or low-level strategy. This strategy, primarily involving subcortical networks, seems to affect visuo-oculomotor activity under the influence of the vestibular error signal and the strong link with proprio-oculomotricity (67), and on the other hand, the vestibular nuclei and the accuracy of the SVV through the recruitment of cervical proprioceptive pathways, especially by the recruitment of COR gain, defined as accuracy in vestibular processing (25, 68). The second system, involving a cognitive or high-level strategy, entails several “possible” compensation mechanisms to influence the control of proprioceptive sensory gain, sensorimotor, cognitive-perceptual, and affective process control (6).

## 5 Conclusion

Our study has highlighted two main points of interest, the first being that of integrative, non-segmented therapy by a panel of paramedical practitioners. Non-pharmacological therapy should not only be responsive to dysfunctions of primary vestibular functions but should also focus on various aspects of visual function and the quality of life of chronic vestibular patients. The significant improvements in near visual acuity, visual fusion, and spatial perception underscore the importance of a real-time strategy in managing vestibular disorders. It is a true somato-perceptual-motor and cognitive-behavioral therapy, these two aspects needing to be merged in care. A second point raised by our study is the notion of new markers that must be systematically questioned before, during, and after therapy, such as neuro-visual and psycho-emotional aspects.

This study also contributes to the discussion in the existing literature (52) which posits the impact of cognitive-vestibular recruitment during compensation tasks on available resources by demonstrating that integrative vestibular rehabilitation can have extensive beneficial effects, positively impacting patients' mental health and quality of life. It underscores the importance of continuing research in this field, particularly to develop more targeted and effective rehabilitation strategies, and to better understand central compensation mechanisms. These efforts will significantly improve the well-being and independence of individuals suffering from chronic vestibular disorders.

## Data Availability

The original contributions presented in the study are included in the article/Supplementary Material, further inquiries can be directed to the corresponding author.
