# Supplementary material for: Innovative approaches for managing patients with chronic vestibular disorders: follow-up indicators and predictive markers for studying the vestibular error signal
Source: Front Rehabil Sci. 2024 Aug 16;5:1414198. doi: 10.3389/fresc.2024.1414198 (PMC11362045; doi:10.3389/fresc.2024.1414198)
Supplement: Supplementary file 1 [file Table1.docx]

Supplementary Material

# SupplementaryTables

**Table 1: The Dizziness Handicap Inventory (DHI)**

| **Scale** | **Number of Items** | **After Recoding, Number of Each Item** |
| --- | --- | --- |
| Physical (P) | 7 | 1, 4, 8, 11, 13, 17, 25 |
| Emotional (E) | 9 | 2, 9, 10, 15, 18, 20, 21, 22, 23 |
| Functional (F) | 9 | 3, 5, 6, 7, 12, 14, 16, 19, 24 |

**Conversion Steps:** Items are recoded according to the following conditions: yes = 4, sometimes = 2, no = 0; the average scores are established by dimension. Finally, each dimension is converted into percentages with maximum scores per dimension as P=28; E=36; F=36.

**Table 2: The Short Form (36) Health Survey (SF36)**

| **Scale** | **Number of Items** | **After Recoding According to Table 1, Average the Following Items** |
| --- | --- | --- |
| Physical Functioning | 10 | Q3a, Q3b, Q3c, Q3d, Q3e, Q3f, Q3g, Q3h, Q3i, Q3j |
| Role Limitations Due to Physical Health | 4 | Q4a, Q4b, Q4c, Q4d |
| Role Limitations Due to Emotional Problems | 3 | Q5a, Q5b, Q5c |
| Energy / Fatigue | 4 | Q9a, Q9e, Q9g, Q9i |
| Emotional Well-being | 5 | Q9b, Q9c, Q9d, Q9f, Q9h |
| Social Functioning | 2 | Q6, Q10 |
| Pain | 2 | Q7, Q8 |
| General Health | 5 | Q1, Q11a, Q11b, Q11c, Q11d |
| Reported Health Transition | 1 | Q2 |

**Conversion Step:** Item recodings are done according to the 1992 interpretation manual. Once the average for each dimension is established, conversion to percentages is performed using the following formula = ((Patient's Mean Raw Score - Lowest Possible Raw Score)) / (Possible Raw Score Range).

Haut du formulaire

**Table 3: Dimensions of the EPN 31 Questionnaire**

| **Emotions** | **Positive Emotions** | **Positive Emotions** | **Positive Emotions** | **Negative Emotions** | **Negative Emotions** | **Negative Emotions** | **Negative Emotions** | **Negative Emotions** | **Surprises** |
| --- | --- | --- | --- | --- | --- | --- | --- | --- | --- |
| **Categories** | JOY | TENDERNESS | TOTAL | FEAR | ANGER | SHAME | SADNESS | TOTAL | TOTAL |
| **Items** | 5, 14, 20, 23, 25 | 1, 2, 4, 29, 30 |  | 3, 18, 21, 22 | 7, 11, 15, 19 | 8, 9, 12, 16, 17, 24 | 6, 10, 26, 31 |  | 13, 27, 28 |

**Reading Key:** After obtaining the totals, convert the scores into percentages.

**Table 4: Big Five Inventory (BFI) Coding and Dimension**

| **Step** | **Description** | **Details** |
| --- | --- | --- |
| Step 1: Recoding Questions | Original Coding | Items: 1, 11, 26, 36, 7, 17, 22, 32, 42, 3, 13, 28, 33, 38, 4, 14, 19, 29, 39, 5, 10, 15, 20, 25, 30, 40, 44 |
|  | Reverse Coding | Items: 6, 16, 21, 31, 2, 12, 27, 37, 45, 8, 18, 23, 43, 9, 24, 34, 35, 41 |
| Step 2: Classification by Dimension | E (Extraversion, Energy, Enthusiasm) | Items: 1, 6R, 11, 16R, 21R, 26, 31R, 36 |
|  | A (Agreeableness, Altruism, Affection) | Items: 2R, 7, 12R, 17, 22, 27R, 32, 37R, 42, 45R |
|  | C (Conscientiousness, Control, Constraint) | Items: 3, 8R, 13, 18R, 23R, 28, 33, 38, 43R |
|  | N (Negative Emotions, Neuroticism, Nervousness) | Items: 4, 9R, 14, 19, 24R, 29, 34R, 39 |
|  | O (Openness, Originality, Open-mindedness) | Items: 5, 10, 15, 20, 25, 30, 35R, 40, 41R, 44 |

**Reading Key:** Reversed Scale : 1 to 5 becomes 5 to 1. "R" indicates reverse-coded items.

**Table 5: VestiQ-VS Questionnaire Dimensions (Vestibular Health Questionnaire)**

| **Dimension** | **Coding** | **Item** |
| --- | --- | --- |
| **Positional Component** | Physical disability detection: YES = 1, NO = 0. A higher score indicates a significant disability in head movement and positional changes. | Benign paroxysmal positional vertigo detection Sequence: 111100000, 111011010, 111010110 |
| **Sensory Disability Assessment** | Rated from 0 to 4: 0 = never, 1 = rarely, 2 = sometimes, 3 = quite often, 4 = very often | 1a, 1b, 2, 3a, 3b, 3c, 3d, 4, 5a, 5b, 5c, 5d, 6, 7, 8, 9, 10 |
| **Daily Life Activity** | Rated from 0 to 4: 0 = never, 1 = rarely, 2 = sometimes, 3 = quite often, 4 = very often, except for item 15 which is RECODED as: 0=4; 1=3; 2=2; 3=1; 4=0 | 11, 12, 13, 14, 15, 16, 17, 18, 19, 20 |
| **Cognition** | Rated from 0 to 4: 0 = never, 1 = rarely, 2 = sometimes, 3 = quite often, 4 = very often | 21, 22, 23, 24 |
| **Psychological Disorders** | Rated from 0 to 4: 0 = never, 1 = rarely, 2 = sometimes, 3 = quite often, 4 = very often | 25, 26, 27, 28, 29 |
| **Emotion** | Rated from 0 to 4: 0 = never, 1 = rarely, 2 = sometimes, 3 = quite often, 4 = very often | 30, 31, 32, 33, 34 |
| **Fatigue** | Rated from 0 to 4: 0 = never, 1 = rarely, 2 = sometimes, 3 = quite often, 4 = very often | 35, 36, 37, 38, 39 |
| **Memory** | Rated from 0 to 4: 0 = never, 1 = rarely, 2 = sometimes, 3 = quite often, 4 = very often | 40, 41, 42, 43, 44 |
| **Executive Functions** | Rated from 0 to 4: 0 = never, 1 = rarely, 2 = sometimes, 3 = quite often, 4 = very often | 45, 46, 47, 48, 49 |
| **Spatial Orientation** | Rated from 0 to 4: 0 = never, 1 = rarely, 2 = sometimes, 3 = quite often, 4 = very often | 50, 51, 52, 53, 54 |

Reading Key: $\boldsymbol{\mu d=}\frac{\sum_{\boldsymbol{i=1}}^{\boldsymbol{n}} \boldsymbol{score}_{\boldsymbol{i}}}{\boldsymbol{nb of items per dimension}}$

$\mu d$ = The average score of the dimension, where n is the number of items within the dimension, and i represents the individual item score, with the number of items per dimension equating to the total number of items within that dimension. The higher the average, the more the evaluated component is affected.

**Table 6: Sensory Organization Test**

| **Condition** | **Support** | **Vision** |
| --- | --- | --- |
| 1 | Static | Eyes open |
| 2 | Static | Eyes closed |
| 3 | Static | Vision tethered |
| 4 | Unstable platform or foam | Eyes open |
| 5 | Unstable platform or foam | Eyes closed |
| 6 | Unstable platform or foam | Vision tethered |

**Table 7: Manufacturer's Norms for the Sensory Organization Assessment (SOA)**

| **Value** | **Somesthetic** | **Visual** | **Vestibular** | **Preferential** | **Composite** | **Romberg Quotient** |
| --- | --- | --- | --- | --- | --- | --- |
| AP | 90 | 84 | 62 | 77 | 69 | [85 ; 241] |
| ML | 97 | 75 | 55 | 74 | 67 | [85 ; 241] |

**Reading Key :**

Somesthetic Score = SE cond SOT 2 / SE cond SOT 1. This is the patient's ability to use their somatosensory system for balance maintenance.

Visual Score = SE cond SOT 4 / SE cond SOT 1. This reflects the patient's ability to utilize visual information for balance.

Vestibular Score = SE cond SOT 5 / SE cond SOT 1. It represents the patient's capability to use their vestibular system for balance.

Preferential Score = (SE cond SOT 3 + SE cond SOT 6) / (SE cond SOT 2 + SE cond SOT 5). It denotes the patient's ability to disregard misleading visual information.

Composite Score. A global score that considers the patient's balancing performance across all sensory conditions.

Romberg Quotient (RQ) = (closed eyes area (CE) / open eyes area (OE)) × 100: a measure of the patient's visual dependency for balance.

**Table 8: Videonystagmography (VNG) Data and Manufacturer's Norms**

| **Exam Phase** | **Test** | **Unit** | **Normative References** |
| --- | --- | --- | --- |
| **Calibration** |  |  |  |
|  | L | ms | < 280 if increase implies nonspecific central involvement |
|  | V | °/s | > 380 if lower: brainstem origin? |
|  | P | % | [80; 105] if asymmetry: cerebellar origin? |
| **Kinetic VNG** |  |  |  |
|  | VVOR | Gain | [0.8; 1.1] and |
|  |  | Prep °/s | < 2 |
|  | VOR, VOR 2 | Gain | [0.4; 1] and |
|  |  | Prep °/s | < 2 |
|  | FOI | Gain | ≤ 0.1 or |
|  |  | Prep °/s | Close to 0 |
|  | COR | Gain | < 0.2 and |
|  |  | °/s | < 2°/s |
| **Thermal VNG** |  |  |  |
|  | AP | °/s | < 2 and |
|  | Reflectivity | °/s | [20; 80] and |
|  | Deficit | % | < 30 |

**Legend:** L: latency; V: velocity; P: precision; °/s: degrees per second; %: percentage; ms: millisecond; VVOR: visuo-vestibular ocular reflex; VOR: vestibulo-ocular reflex; VOR 2: vestibulo-ocular reflex during dual-task; OFI: ocular fixation index; AP: absolute predominance

**Table 9: Statistical Analysis Results of Study Questionnaires**

| **Variable** | **Mean** | **p-value** |
| --- | --- | --- |
| DHI P1 : Physical dimension before iTRV | 60.96 | 3.74E-08*** |
| DHI P2 : Physical dimension after iTRV | 36.35 |  |
| DHI E1 : Emotional dimension before iTRV | 45.31 | 2.69E-06*** |
| DHI E2 : Emotional dimension after iTRV | 28.57 |  |
| DHI F1 : Functional dimension before iTRV | 50.00 | 2.22E-07*** |
| DHI F2 : Functional dimension after iTRV | 29.17 |  |
| SF36 FP1 : Physical Functioning dimension before iTRV | 62.41 | 0.0002*** |
| SF36 FP2 : Physical Functioning dimension after iTRV | 76.34 |  |
| SF36_limitSP1: Role Limitations Due to Physical Health dimension before iTRV | 68,10 | 0,0043*** |
| SF36_limitSP2: Role Limitations Due to Physical Health dimension after iTRV | 52,68 |  |
| SF36_limitE1: Role Limitations Due to Emotional Problems dimension before iTRV | 57,47 | 0,0154** |
| SF36_limitE2: Role Limitations Due to Emotional Problems dimension after iTRV | 40,48 |  |
| SF36_NRJ1: Energy / Fatigue dimension before iTRV | 42,16 | 0,0007*** |
| SF36_NRJ2: Energy / Fatigue dimension after iTRV | 50,63 |  |
| SF36_BEE1: Emotional Well-being dimension before iTRV | 50,55 | 0,0055** |
| SF36_BEE2: Emotional Well-being dimension after iTRV | 58,07 |  |
| SF36_foncSo1: Social Functioning dimension before iTRV | 68,10 | 0,0011*** |
| SF36_foncSo2: Social Functioning dimension after iTRV | 78,13 |  |
| SF36_doul1: Pain dimension before iTRV | 77,12 | 0,1803 |
| SF36_doul2: Pain dimension after iTRV | 81,61 |  |
| SF36_SG1: General Health dimension before iTRV | 49,33 | 0.0193** |
| SF36_SG2: General Health dimension after iTRV | 56,14 |  |
| EPN_JOIE1: Joy dimension before iTRV | 19,69 | 3.39e-07*** |
| EPN_JOIE2: Joy dimension after iTRV | 23,80 |  |
| EPN_TENDR1: Tenderness dimension before iTRV | 25,81 | 0,0141** |
| EPN_TENDR2: Tenderness dimension after iTRV | 27,98 |  |
| EPN_PEUR1: Fear dimension before iTRV | 18,00 | 0,0417* |
| EPN_PEUR2: Fear dimension after iTRV | 15,25 |  |
| EPN_COLER1: Anger dimension before iTRV | 11,93 | 0,1380 |
| EPN_COLER2: Anger dimension after iTRV | 10,88 |  |
| EPN_HONT1: Shame dimension before iTRV | 12,09 | 0,0186** |
| EPN_HONT2: Shame dimension after iTRV | 10,73 |  |
| EPN_TRIST1: Sadness dimension before iTRV | 14,90 | 0.0577* |
| EPN_TRIST2: Sadness dimension after iTRV | 13,52 |  |
| EPN_SURPRI1: Surprise dimension before iTRV | 7,21 | 0,1240 |
| EPN_SURPRI2: Surprise dimension after iTRV | 7,73 |  |
| BFI_EEEA1: (Extraversion, Energy, Enthusiasm) dimension before iTRV | 3,05 | 0,1545 |
| BFI_EEEA2: (Extraversion, Energy, Enthusiasm) dimension after iTRV | 2,87 |  |
| BFI_AAAA1: (Agreeableness, Altruism, Affection) dimension before iTRV | 4,22 | 0,1752 |
| BFI_AAAA2: (Agreeableness, Altruism, Affection) dimension after iTRV | 3,99 |  |
| BFI_CCCA1: Conscientiousness, Control, Constraint) dimension before iTRV | 4,00 | 0,6721 |
| BFI_CCCA2: Conscientiousness, Control, Constraint) dimension after iTRV | 3,86 |  |
| BFI_ENNA1: (Negative Emotions, Neuroticism, Nervousness) dimension before iTRV | 3,16 | 0,0059* |
| BFI_ENNA2: (Negative Emotions, Neuroticism, Nervousness) dimension after iTRV | 2,76 |  |
| BFI_OOOA1: (Openness, Originality, Open-mindedness) dimension before iTRV | 3,29 | 0,3996 |
| BFI_OOOA2: (Openness, Originality, Open-mindedness) dimension after iTRV | 3,15 |  |
| VESTIPOA1: Positional Component dimension before iTRV | 51,52 | 1,05E-07*** |
| VESTIPOA2: Positional Component dimension after iTRV | 23,84 |  |
| VESTIANA1: Sensory Disability Assessment dimension before iTRV | 39,87 | 2,91E-10*** |
| VESTIANA2: Sensory Disability Assessment dimension after iTRV | 18,88 |  |
| VESTBQA1: Daily Life Activity dimension before iTRV | 57,92 | 1,79E-08*** |
| VESTIAVQA2: Daily Life Activity dimension after iTRV | 30,05 |  |
| VESTICOGA1: Cognition dimension before iTRV | 42,25 | 0,0008*** |
| VESTICOGA2: Cognition dimension after iTRV | 27,73 |  |
| VESTPSYA1: Psychological Disorders dimension before iTRV | 47,31 | 3,70E-06*** |
| VESTIPSYA2: Psychological Disorders dimension after iTRV | 27,64 |  |
| VESTIEMOA1: Emotion dimension before iTRV | 39,07 | 0,0002*** |
| VESTIEMOA2: Emotion dimension after iTRV | 24,55 |  |
| VESTIFATA1: Fatigue dimension before iTRV | 51,02 | 0,0022*** |
| VESTIFATA2: Fatigue dimension after iTRV | 39,82 |  |
| VESTIMEMA1: Memory dimension before iTRV | 28,61 | 0,7596 |
| VESTMEMA2: Memory dimension after iTRV | 30,36 |  |
| VESTFEA1: Executive Functions dimension before iTRV | 45,56 | 0,0005*** |
| VESTFEA2: Executive Functions dimension after iTRV | 30,36 |  |
| VESTIAPTA1: Spatial Orientation dimension before iTRV | 29,54 | 0,1115 |
| VESTIAPTA2: Spatial Orientation dimension after iTRV | 25,45 |  |

**Legend:** * trend towards statistical significance (p-value >0.05), ** moderate statistical significance (0.01 < p-value ≤ 0.05),

*** strong statistical significance (p-value ≤ 0.01)

**Table 10: Normality Study of Variables from the Subjective Visual Vertical Analysis**

| **Variable** | **Population** | **Statistic** | **p-value** | **Normality Status** |
| --- | --- | --- | --- | --- |
| STATIC GEOMETRIC ANGLE A1 | I | 0.85 | <0.01*** | AN |
| STATIC GEOMETRIC ANGLE A1 | D | 0.95 | 0.28 | N |
| STATIC GEOMETRIC ANGLE A1 | Total | 0.90 | <0.01*** | AN |
| STATIC BISECTOR ANGLE A1 | I | 0.99 | 0.95 | N |
| STATIC BISECTOR ANGLE A1 | D | 0.94 | 0.23 | N |
| STATIC BISECTOR ANGLE A1 | Total | 0.99 | 0.99 | N |
| DYNAMIC GEOMETRIC ANGLE A1 | I | 0.93 | 0.05** | N |
| DYNAMIC GEOMETRIC ANGLE A1 | D | 0.94 | 0.10* | N |
| DYNAMIC GEOMETRIC ANGLE A1 | Total | 0.95 | 0.01*** | AN |
| DYNAMIC BISECTOR ANGLE A1 | I | 0.96 | 0.20 | N |
| DYNAMIC BISECTOR ANGLE A1 | D | 0,96 | 0,56 | N |
| DYNAMIC BISECTOR ANGLE A1 | Total | 0,97 | 0,14 | N |
| STATIC GEOMETRIC ANGLE A2 | I | 0,98 | 0,88 | N |
| STATIC GEOMETRIC ANGLE A2 | D | 0,97 | 0,50 | N |
| STATIC GEOMETRIC ANGLE A2 | Total | 0,96 | 0,04** | AN |
| STATIC BISECTOR ANGLE A2 | I | 0,92 | 0,01** | AN |
| STATIC BISECTOR ANGLE A2 | D | 0,94 | 0,28 | N |
| STATIC BISECTOR ANGLE A2 | Total | 0,98 | 0,40 | N |
| DYNAMIC GEOMETRIC ANGLE A2 | I | 0,91 | 0,01** | AN |
| DYNAMIC GEOMETRIC ANGLE A2 | D | 0,94 | 0,09* | N |
| DYNAMIC GEOMETRIC ANGLE A2 | Total | 0,95 | 0,01** | AN |
| DYNAMIC BISECTOR ANGLE A2 | I | 0,93 | 0,01** | AN |
| DYNAMIC BISECTOR ANGLE A2 | D | 0,95 | 0,33 | N |
| DYNAMIC BISECTOR ANGLE A2 | Total | 0,97 | 0,23 | N |

**Legend:** * trend towards statistical significance (p-value >0.05), ** moderate statistical significance (0.01 < p-value ≤ 0.05),

*** strong statistical significance (p-value ≤ 0.01). A1: Measurement before hTRV, A2: Measurement after hTRV, N : Normal, AN : Abnormal status of the SSV, I : Improvement, D : Deterioration.

**Table 11: Distribution of Variation States by Variable, Group, and Type of Angle from A1 to A2**

| **Group** | **Group Size** | **State** | **Static VVS Geometric Angle**  **CV (Cg)** | **Static VVS Bisecting Angle**  **CV (Cg)** | **Dynamic VVS Geometric Angle**  **CV (Cg)** | **Dynamic VVS Bisecting Angle**  **CV (Cg)** |
| --- | --- | --- | --- | --- | --- | --- |
| **Variable Hyperactive Signal** | | | | | | |
| **Absent** | 55 | Improvement | 31 (56%) | 23 (42%) | 30 (55%) | 33 (60%)* |
|  |  | Deterioration | 24 (44%) | 32 (58%)* | 25 (45%) | 19 (35%) |
| **Present** | 7 | Improvement | 5 (71%) | 5 (71%)* | 2 (29%) | 5 (71%)* |
|  |  | Deterioration | 2 (29%) | 2 (29%) | 5 (71%)* | 2 (29%) |
| **Variable EdC** | | | | | | |
| **Stable** | 42 | Improvement | 23 (55%) | 20 (48%) | 26 (62%) | 27 (64%)* |
|  |  | Deterioration | 19 (45%) | 22 (52%) | 16 (38%) | 15 (36%) |
| **Unstable** | 18 | Improvement | 12 (67%)* | 7 (39%) | 6 (33%) | 9 (50%) |
|  |  | Deterioration | 6 (33%) | 11 (61%)* | 12 (67%)* | 6 (33%) |
| **Variable COR Gain** | | | | | | |
| **Increase** | 25 | Improvement | 14 (56%) | 14 (56%) | 15 (60%)* | 12 (48%) |
|  |  | Deterioration | 11 (44%) | 11 (44%) | 10 (40%) | 10 (40%) |
| **Stable** | 5 | Improvement | 4 (80%)* | 2 (40%) | 2 (40%) | 4 (80%)* |
|  |  | Deterioration | 1 (20%) | 3 (60%) | 3 (60%) | 1 (20%) |
| **Decrease** | 31 | Improvement | 17 (55%) | 11 (35%) | 14 (45%) | 12 (39%) |
|  |  | Deterioration | 14 (45%) | 20 (65%)* | 17 (55%) | 10 (32%) |

**Legend:** * trend towards statistical significance (p-value >0.05), ** moderate statistical significance (0.01 < p-value ≤ 0.05),

*** strong statistical significance (p-value ≤ 0.01). A1: Measurement before hTRV, A2: Measurement after hTRV, EdC: State of compensation assessed through measurements made with bithermal VNG (Videonystagmography)

**Table 12: Results from the Analysis Obtained at the Mawas Board (PmW)**

| **Test distance in centimeters** | **McNemar's chi-squared** | **dF** | **p-value** |
| --- | --- | --- | --- |
| 5 | 0,00 | 1 | 1,00 |
| 10 | 0,50 | 1 | 0,48 |
| 15 | 3,70 | 1 | 0,05* |
| 20 | 6,86 | 1 | 0,01*** |
| 25 | 2,78 | 1 | 0,10 |
| 30 | 1,89 | 1 | 0,17 |
| 35 | 2,56 | 1 | 0,11 |
| 40 | 0,04 | 1 | 0,84 |

**Legend:** * trend towards statistical significance (p-value >0.05), ** moderate statistical significance (0.01 < p-value ≤ 0.05),

*** strong statistical significance (p-value ≤ 0.01)

**Table 13: Results from the Analysis of Measurements of Near Points of Accommodation (NPA) and Near Point of Convergence (NPC)**

| **Test distance in meters** | **McNemar's chi-squared** | **dF** | **p-value** |
| --- | --- | --- | --- |
| Circle 1m | 2,77 | 1 | 0,10 |
| Star 1m | 5,26 | 1 | 0,02** |
| Cat 1m | 3,12 | 1 | 0,08 |
| Car 1m | 5,33 | 1 | 0,02** |
| Circle 5m | 5,06 | 1 | 0,02** |
| Star 5m | 4,08 | 1 | 0,04** |
| Cat 5m | 0,36 | 1 | 0,55 |
| Car 5m | 0,36 | 1 | 0,55 |

**Legend:** * trend towards statistical significance (p-value >0.05), ** moderate statistical significance (0.01 < p-value ≤ 0.05),

*** strong statistical significance (p-value ≤ 0.01)
